# Preliminary Discovery of Small Molecule Inhibitors of Epidermal Growth Factor Receptor (EGFR) That Bind to the Extracellular Domain

**DOI:** 10.3390/cancers14153647

**Published:** 2022-07-27

**Authors:** Rosa Di Liddo, Marco Verona, Christian Vaccarin, Laura Acquasaliente, Sandra Schrenk, Monica Piccione, Carola Cenzi, Michele De Franco, Matteo Dal Prà, Giovanni Ribaudo, Maria Grazia Ferlin, Maria Teresa Conconi, Adriana Chilin, Valentina Gandin, Giovanni Marzaro

**Affiliations:** 1 Department of Pharmaceutical and Pharmacological Sciences, University of Padova, Via Marzolo 5, I-35131 Padova, Italy; rosa.diliddo@unipd.it (R.D.L.); marco.verona@phd.unipd.it (M.V.); christian.vaccarin@phd.unipd.it (C.V.); laura.acquasaliente@unipd.it (L.A.); sandra89.schrenk@gmail.com (S.S.); monica.piccione@studenti.unipd.it (M.P.); carola.cenzi@iov.veneto.it (C.C.); miky_defra@hotmail.com (M.D.F.); matteo.dal-pra@autifony.com (M.D.P.); mariagrazia.ferlin@unipd.it (M.G.F.); mariateresa.conconi@unipd.it (M.T.C.); adriana.chilin@unipd.it (A.C.); valentina.gandin@unipd.it (V.G.); 2 Center for Radiopharmaceutical Sciences, ETH-PSI-USZ, Paul Scherrer Institute, 5232 Villigen, Switzerland; 3 Department of Molecular and Translational Medicine, University of Brescia, Viale Europa 11, I-25123 Brescia, Italy; giovanni.ribaudo@unibs.it

**Keywords:** EGFR-ECD, trafficking, inhibition, isatin

## Abstract

**Simple Summary:**

The Epidermal Growth Factor Receptor (EGFR) is a receptor protein involved in many types of cancers. EGFR can be inhibited by monoclonal antibodies (protein drugs acting on the extracellular domain of the protein) or by ATP-mimic compounds (small molecule drugs blocking the intracellular domain). Here we report the identification of a novel potential class of drugs, i.e., small molecules acting on the extracellular domain of EGFR. The identified compounds modified the trafficking of EGFR and induced cytotoxicity in cells overexpressing EGFR and insensitive to monoclonal antibodies being active in cell lines bearing KRAS mutations.

**Abstract:**

The Epidermal Growth Factor Receptor (EGFR) is a transmembrane glycoprotein belonging to the protein kinase superfamily. It is composed of an extracellular domain, a transmembrane anchoring region and a cytoplasmic region endowed with tyrosine kinase activity. Genetic mutations of EGFR kinase cause higher activity thereby stimulating downstream signaling pathways that, in turn, impact transcription and cell cycle progression. Due to the involvement of mutant EGFR in tumors and inflammatory diseases, in the past decade, several EGFR inhibitory strategies have been extensively studied, either targeting the extracellular domain (through monoclonal antibodies) or the intracellular kinase domain (through ATP-mimic small molecules). Monoclonal antibodies impair the binding to growth factor, the receptor dimerization, and its activation, whereas small molecules block the intracellular catalytic activity. Herein, we describe the development of a novel small molecule, called **DSF-102,** that interacts with the extracellular domain of EGFR. When tested in vitro in KRAS mutant A549 cells, it impairs EGFR activity by exerting (i) dose-dependent toxicity effects; (ii) a negative regulation of ERK, MAPK p38 and AKT; and (iii) a modulation of the intracellular trafficking and lysosomal degradation of EGFR. Interestingly, **DSF-102** exerts its EGFR inhibitory activity without showing interaction with the intracellular kinase domain. Taken together, these findings suggest that **DSF-102** is a promising hit compound for the development of a novel class of anti-EGFR compounds, i.e., small molecules able to interact with the extracellular domain of EGFR and useful for overcoming the KRAS-driven resistance to TKI treatment.

## 1. Introduction

**Figure 1:**
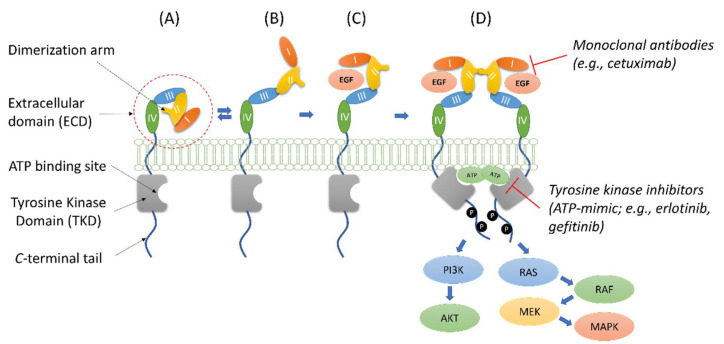
Structure and simplified dynamics representation of EGFR. The auto-tethered fully inactive conformation of EGFR (**A**) is in equilibrium with a partially opened state (**B**) that can be stabilized by the binding with EGF (**C**). The EGF-activated conformation can dimerize (**D**) leading to receptor autophosphorylation and activation of the AKT and MAPK cascades that mediate cell survival and proliferation. The target regions for monoclonal antibodies and ATP-mimic compounds are also reported in (**D**).

The Epidermal Growth Factor Receptor (EGFR) is a transmembrane Receptor Tyrosine Kinase (RTK) involved in the regulation of cell growth, differentiation, apoptosis, and gene transcription. The structure and functions of EGFR (Figure 1) are well-known and characterized, since EGFR is among the first studied RTKs [1,2].

EGFR comprises three domains: (i) an extracellular domain (EGFR-ECD) deputed to the binding with growth factor ligands (e.g., EGF, TGFα); (ii) a transmembrane region; (iii) an intracellular tyrosine kinase domain (EGFR-TKD). EGFR-ECD comprises four sub-units (domains I–IV). Domain II contains the dimerization arm and mediates the receptor dimerization; domains I and III serve for the interaction with the growth factors. EGFR-ECD can adopt different conformations, as revealed by both experimental studies and molecular dynamic simulations [3,4,5]. A very simple depiction of the EGFR-ECD behavior can be given by considering two limit situations: (i) the auto-tethered conformation, characterized by the interaction of domains II and IV, with the dimerization arm completely occluded and unable to dimerize; (ii) the extended conformation, characterized by the exposure of the dimerization arm. Although the auto-tethered and the extended conformations are in equilibrium, the exposure of dimerization arm is not sufficient by itself for receptor dimerization, and the binding with the growth factors is essential for inducing the receptor activation [6]. Once the dimerization occurs, the intracellular kinase domains undergo auto(trans)phosphorylation, initiating the signal transduction. Downstream signals promoted by EGFR involve different pathways, comprising the PI3K/AKT route, the MAPK way and the phospholipase-C signaling [7]. To prevent an excessive EGFR activation, a strong down-regulation of the receptor is required, which involves EGFR intracellular transport [7,8,9]. The EGFR trafficking is influenced by the extracellular EGF concentration [10]: under low EGF concentration, clathrin-mediated endocytosis is mainly activated, and a large part of internalized EGFR is recycled back to the plasma membrane. Higher EGF concentration increases the ratio of lysosomal degradation. The ligand-activated EGFR signaling controls unliganded receptors through a feedback phosphorylation, identifying a dual-mode regulation of the endocytic dynamics of EGFR [10]. A minimal activation of EGFR by low EGF concentration activates downstream MAPK pathways that efficiently phosphorylate the remaining unbound EGFR molecules. This non-canonical regulation of EGFR involves the phosphorylation, mediated by p38, of EGFR at its *C*-tail and promotes the endocytosis, mediated by the clathrin, of EGF-free EGFR monomers. Moreover, the non-canonical regulation has a different timeline than the canonical endocytosis (i.e., the internalization of EGF-bound EGFR dimers): the former, indeed, occurs later than the latter. At high EGF concentration, approximately half of EGFR endocytosis is reported to be clathrin-independent. In this condition, p38-mediated phosphorylation cannot occur, and EGFR dimers undergo to lysosomal degradation [10,11]. Within a few hours of EGF stimulation, EGFR can also translocate to the nucleus, where it promotes gene transcription, DNA repair, and radio- and chemo-resistance [12]. EGFR acts as an indirect transcription regulator: although it does not contain any DNA-binding site, it was reported to stimulate the transcription of different cancer promoting genes [13,14]. Abnormal EGFR activity leads to an excess of proliferative signaling and the role of EGFR hyperactivation in cancer onset and progression has been demonstrated for different types of cancer [15]. The inhibition of EGFR can be accomplished either by blocking the kinase activity (using ATP-mimic drugs, TKIs, targeting the EGFR-TKD, as gefitinib or erlotinib) or by impairing the binding with EGF (through monoclonal antibodies, mAbs, targeting the EGFR-ECD as cetuximab) [7]. The high cost of production and the impossibility to adopt an oral administration are two major drawbacks related to the use of mAbs. Conversely, TKIs must be able to cross the cell membrane and to be insensitive to efflux pumps to reach the kinase domain at a pharmacologically relevant concentration.

The discovery of orally available small molecules able to impair the EGFR activation targeting the extracellular domain could be of great interest for cancer treatment. To pursue this goal, a suitable high/medium throughput screening method is required. In this respect, a commercially available kit (PathHunter^®^ from DiscoverX©; San Francisco, CA, USA) for the screening of compounds able to prevent EGFR activation (either dimerization or autophosphorylation) has been developed in recent years. This assay measures the chemiluminescent signal produced as a consequence of ligand-induced dimerization/activation of EGFR [16]. Our interest in kinase inhibitors [17,18,19] and the readily availability of such kit prompted us to start a campaign for the discovery of small molecules targeting the extracellular domain of EGFR. Recently, Yao et al. reported the discovery of **DPBA**, the first EGFR-ECD small molecule able to suppress the EGFR activation, to block the dimerization and to induce the protein internalization and degradation [20], further demonstrating the feasibility of this goal. **DPBA** bound the EGFR with micromolar affinity (Kd = 38.4 ± 1.8 μM) and no other analogues of the compound were reported in the paper.

Herein we report the preliminary discovery of a new class of EGFR-ECD binders and the development of a novel small molecule (**DSF-102**) able to hamper the EGFR-mediated signal transduction by binding the EGFR-ECD and impairing the interaction with the EGF instead of blocking the intracellular kinase activity. Moreover, as increasing evidence shows that KRAS mutations correlate with unresponsiveness to EGFR inhibitors [21], we tested the biological activity of **DSF-102** in a lung adenocarcinoma cell line, the A549 KRAS mutant cells. Of note, A549 cells are partly resistant to cetuximab (the only pharmacological agent targeting EGFR-ECD so far approved) treatment. Indeed, cetuximab was shown to inhibit the pathways involved in cell proliferation, cell survival and tumor invasion (i.e., the RAS–RAF–mitogen-activated protein kinase 1 (MAPK1) and the v-AKT murine thymoma viral oncogene homolog 1 (AKT1) axis) in cells overexpressing EGFR but not in KRAS-mutated tumors. The comparison of biological activity of **DSF-102** with cetuximab in A549 cells in terms of induction of apoptosis, effects on MAPKs phosphorylation and receptor internalization gave, thus, preliminary information on the ability of our new compound to overcome the KRAS-mediated resistance to anti-EGFR treatment.

## 2. Materials and Methods

### 2.1. Chemistry

Solvents and chemicals were used as provided by the suppliers (Carlo Erba and Sigma Aldrich). A CEM Discover monomode reactor was used for the microwave-assisted organic synthesis. The microwave was supplied with an infrared sensor for the temperature monitoring and with the automatic control of the irradiation power. All the reactions were run in a closed vial. Thin layer chromatography (TLC) was conducted on pre-coated plates (silica gel 60; 0.2 mm; fluorescent indicator UV254; supplier: Merck), while for column chromatography silica gel 60 (0.063–0.100 mm; supplier: Merck) was used. The ^1^H-NMR and ^13^C-NMR spectra were determined on a Bruker 300 spectrometer; chemical shifts are reported in ppm and coupling constants are given in Hz. The purity of the compounds was checked by HPLC using a Shimadzu LC-10 AT-VP instrument equipped with an UV detector (wavelength for detection = 254 nm). The analyses were performed with a flowrate of 1 mL/min and C18 column (250 × 4.6 mm, 5 nm particle size). The mobile phase consisted of phase A (milli-Q water, 18.0 MU, TFA 0.05%) and phase B (acetonitrile). Gradient elution was performed as follows: 0 min, %B = 10; 20 min, %B = 90; 25 min, %B = 90; 26 min, %B = 10; 31 min, %B = 10. All compounds are >95% pure by HPLC analysis and ^1^H-NMR spectra. A representative HPLC chromatogram as well as ^1^H- and ^13^C-NMR for the most representative compound (**DSF-102**) have been reported in the Supplementary Materials (Figures S3–S5).

The synthesis of compounds **DSF-102** is reported here in details. The synthesis of all other analogues (**DSF-067/-070** and **DSF-091/-103**) are reported in Supporting Information.

#### 2.1.1. Synthesis of 2-(*N*-hydroxylamino)-*N*-(4-nitrophenyl)acetamide (**15**)

A solution of chloral hydrate (1.82 g, 11.0 mmol) and anhydrous sodium sulfate (12.5 g, 88.0 mmol) in water (25 mL) was added to a solution of 4-nitroaniline **7** (1.38 g, 10.0 mmol) in 1M aqueous hydrochloric acid (10 mL) and to a solution of hydroxylamine hydrochloride (2.37 g, 34.0 mmol) in water (10 mL). The mixture was heated at 80 °C for two hours and then cooled to room temperature. The solid precipitate was collected by vacuum filtration, washed with cold water, and dried, yielding compounds **15** (yield: 83%). Analytical data agreed with literature [22].

#### 2.1.2. Synthesis of 5-Nitroindolin-2,3-dione (**23**)

Compound **15** (1.05 g, 5.0 mmol) was suspended in 15 mL conc. sulfuric acid. The mixture was heated at 60 °C for 15 min, cooled to room temperature and then poured into ice water (150 mL). The obtained precipitate was filtered and washed extensively with water, furnishing compound **23** (yield 62%). Analytical data agreed with literature [22].

#### 2.1.3. Synthesis of (*E*,*Z*)-3-(3-(Trifluoromethyl)benzylidene)-5-nitroindolin-2-one (**DSF-102**)

Compound **23** (0.19 g, 1.0 mmol) and 3-aminobenzotrifluoride (0.12 mL, 1.0 mmol) were suspended in ethanol (3 mL). The reaction mixture was added of glacial acetic acid (100 µL) and irradiated under microwave (200 W, 115 °C) for 30 min. After cooling to room temperature, the obtained precipitate was filtered and washed with ethanol. Yield 27.8%.

Ratio E/Z: 65:35; mp = 250–252 °C; IR (KBr): ṽ (cm^−1^) = 3297 (NH), 1751 (C=O), 1618 (C=N), 1328 (C-F), 1164 (C-O), 1125 (N=O) cm^−1^.; HRMS (ESI-TOF) for C15H9F3N3O3 (M+H)+: calcd = 336.0591, found = 336.0579.

Isomer E: ^1^H NMR (300 MHz, DMSO-*d_6_*) δ 8.30 (dd, J = 8.8, J = 2.3, 1H), 7.78 (t, J = 7.8, 1H), 7.72 (d, J = 7.8, 1H), 7.48 (s, 1H), 7.39 (d, J = 7.8, 1H), 7.12–7.07 (m, 2H); ^13^C NMR (75 MHz, DMSO-*d_6_*) δ 165.4, 155.8, 150.9, 141.4, 139.7, 131.6, 131.0, 130.9, 124.6, 122.4, 122.0, 120.7, 114.8, 112.7. Isomer Z: ^1^H NMR (300 MHz, DMSO-*d_6_*) δ 8.40 (dd, J = 8.7, J = 2.5, 1H), 8.27 (d, J = 2.5, 1H), 7.58 (t, J = 7.9, 1H), 7.51 (d, J = 7.9, 1H), 7.48–7.46 (m, 1H), 7.36 (d, J = 7.9, 1H), 7.10–7.06 (m, 1H); ^13^C NMR (75 MHz, DMSO-*d_6_*) δ 160.6, 155.5, 149.7, 142.5, 137.5, 130.9, 130.8, 129.7, 124.6, 124.4, 123.0, 121.7, 118.3, 116.3, 111.9.

### 2.2. Biology

#### 2.2.1. Inhibition of EGFR Dimerization

The inhibition of the EGFR dimerization assay was conducted using the PathHunter kit following the producer instructions and with cetuximab as a positive control. The screening results are reported in Table S1. For IC_50_ determination, the selected compounds were tested at different concentration (range 0.1–100 µM) and the IC_50_ values were calculated by the four parameters logistic (4-PL) model. Evaluation was based on means from at least four independent experiments.

#### 2.2.2. Inhibition of EGFR Kinase Activity

The inhibition of the kinase activity was assessed through the KinomeScan assay service (DiscoverX) which is run in parallel with known inhibitors. An exhaustive description of the assay is given in ref. [23] 

#### 2.2.3. Surface Plasmonic Resonance (SPR) Experiments

SPR measurements were carried out at 25 °C on a dual flow-cell Biacore-X100 instrument (GE-Healthcare, Chicago, IL, USA). EGFR-ECD (SinoBiological, Beijing China) was immobilized (3360 RU) on a carboxymethylated-dextran chip (CM5), using the amide coupling chemistry at pH 4.0. Increasing concentrations of recombinant EGF (DiscoverX Inc., San Francisco, USA) were injected over the EGFR-coated sensor chip at a flow rate of 10 µL/min, with a contact time of 180 s, in 10 mM Hepes pH 7.4, 150 mM NaCl, 3 mM EDTA, 0.005% polyoxyethylene sorbitan. See Supporting Information for a detailed description of the experiments.

#### 2.2.4. Cell Culture

The biological activity of **DSF-102** was evaluated in vitro using human epithelial lung cancer A549 cell line, human triple negative breast MDA-MB-231, human colon HCT-15 cancer cells, expressing high levels of wild-type EGFR and endowed with KRAS mutations [21,24,25], as well as human melanoma A375 cells, poorly expressing EGFR and with WT KRAS [26]. Cells were seeded (1 × 10^4^ cells/cm^2^) in tissue culture dishes (BD Falcon, Milan, Italy) and cultured in the following reported appropriate medium supplemented with 10% foetal bovine serum (FBS; Sigma Aldrich, Darmstadt, Germany) and 1% penicillin/streptomycin (Invitrogen, Darmstadt, Germany), under standard conditions [37 °C, 95% humidity, 5% CO_2_]: (i) DMEM/F12 medium for A549 cells (Sigma Aldrich, St. Louis, MO, USA); (ii) RPMI-1640 for MDA-MD-231 and HCT-15 cells; (iii) DMEM for A375 cells. A549, MDA-MB-231, HCT-15, A375 and primary normal dermal fibroblasts (NDFa) cells were purchased from the American Type Culture Collection (ATCC, Manassas, VA, USA). On reaching 70% confluence, the A549 cell cultures were treated with **DSF-102** (2 µM or 50 µM) in medium containing 2% FBS. In parallel, controls were obtained treating cells with cetuximab (10 µg/mL; 0.07 µM) or keeping samples under resting conditions. Moreover, in order to detect potential off-target effects, **DSF-102** was tested on primary normal dermal fibroblasts (NDFa) cultured in DMEM-F12 (Sigma Aldrich) supplemented with 10% FBS (Sigma Aldrich) and 1% penicillin/ streptomycin (Invitrogen, Darmstadt, Germany), under standard conditions (37 °C, 95% humidity, 5% CO_2_). At different time points, cells were collected for the analysis of cell death, AKT signaling activation, endocytic trafficking, membrane EGFR, and cytoplasmic EGF expression. The effect of **DSF-102** on p44/42 ERK and p38 MPK was evaluated in samples co-administered with EGF (100 ng/mL).

#### 2.2.5. Cell Viability

At 24 h from treatment, cell viability was studied by optical microscopy and flow cytometry. The detection of apoptotic cells was performed using BD Cell Viability Kit (BD Biosciences, Milan, Italy) according to the manufacturer’s instructions. After incubation with 2.0 mL of thiazole orange (TO), a nucleic-acid-specific dye, and 1.0 mL of propidium iodide (PI) at room temperature (RT) for 5 min, protected from light, stained cells were analyzed using a fluorescence-activated cell sorter flow cytometer (BD FACSCanto^TM^ II system, Becton Dickinson, San Jose, CA, USA) equipped with BD FACSDiva software. For each analysis, experiments were performed in triplicate and 1 × 10^4^ cells were used. Statistical significance was calculated by Student’s t-test comparing **DSF-102**-treated cultures to controls. 

#### 2.2.6. MTT Assay

The growth inhibitory effect toward MDA-MB-231, HCT-15 and A375 cells was evaluated by means of MTT assay. Briefly, 5–8 × 10^3^ cells/well, dependent upon the growth characteristics of the cell line, were seeded in 96-well microplates in fresh culture medium (100 μL). After 24 h, the medium was removed and replaced with growth medium containing increasing concentrations of **DSF-102**. Each treatment was run with triplicate cultures. Each well was added of MTT (10 µL of 5 mg/mL saline solution) after 24 h and left in incubation for additional 5 h. Then, sodium dodecylsulfate (SDS; 100 µL of 0.01 M HCl solution) was added. The wells were left to incubate overnight, and the cell growth inhibition was detected with a microplate reader (Bio-Rad 680) by measuring the absorbance at 570 nm. The data were expressed as percentage of the untreated control vs. drug concentration. A four-parametric logistic model was used to compute the IC_50_ values (i.e., the concentration of drugs that reduced the mean absorbance at 570 nm to 50% with respect to the untreated control). All the values are expressed as the mean ± SD of at least three independent experiments. 

#### 2.2.7. Immunofluorescence

Controls and **DSF-102**–treated cells were fixed with BD Cytofix solution (BD Biosciences) according to the manufacturer’s instructions. To detect intracellular target proteins, a treatment with 0.5% Triton solution (Sigma Aldrich) was performed at RT. Unspecific sites were blocked using 1% bovine serum albumin (BSA) (Sigma Aldrich) solution in PBS before incubation at 4 °C for 1 h with rabbit anti-human EGFR, mouse anti-human EEA1, -Rab 7, -LAMP1 (Santa Cruz Biotechnology, Inc., Dallas, TX, USA). After washing in PBS, all samples were treated with anti- mouse Alexa Fluor^®^ 594- (Santa Cruz Biotechnology, Inc., Santa Cruz, CA, USA) or anti-rabbit Alexa Fluor^®^ 488-conjugated secondary antibody (Invitrogen, Waltham, MA, USA) at 4 °C for 1 h. In parallel, samples treated with only secondary antibodies were used as reference. After mounting with Fluoro-gel II solution containing DAPI (Electron Microscopy Sciences, Hatfield, PA, USA), all samples were analyzed using Leica SP5 TCS confocal microscope (Leica, Wetzlar, Germany). Furtherly, the investigation of EGFR lysosomal degradation was performed by indirect staining with primary antibodies against EGFR and LAMP1 in samples co-treated with EGF (100 ng/mL) and **DSF-102** for 60 min. To exclude the surface ligand binding EGF from analysis, samples were submitted to acid washing in 150 mM NaCl and 20 mM HCl, pH 1.7, for 3 min at room temperature, as reported by Karagiannis et al. [27]. 

#### 2.2.8. Flow Cytometry

Clinical trials using first-generation EGFR TKIs demonstrated limited benefits to patients affected by KRAS mutations that are currently considered to be biomarkers to exclude patients for EGFR TKI therapy [28,29,30]. To examine the action of **DSF-102** on EGFR in KRAS mutant cells, we treated A549 cells with low (2 µM) and high (50 µM) concentration for 15, 30, 60 min and the phosphorylation level of AKT1, which is a downstream molecule in the EGFR signaling pathway, was evaluated by flow cytometry. Controls were obtained using samples treated with cetuximab (10 µg/mL; 0.07 µM) or resting cells. Moreover, to verify that **DSF-102** molecule affects membrane EGFR content and cytoplasmic EGF, the cells were treated for 1 h with BD Golgi Plug ^TM^ Protein Transport Inhibitor (brefeldin A, BFA) alone or combined with **DSF-102** (BFA/**DSF-102**). The analysis was performed by indirect staining in cells cultured for 1 h under standard or primed conditions with BFA and/or **DSF-102**. After washing with 0.5% BSA in PBS, the samples were incubated with primary antibodies (rabbit anti-human EGFR (Santa Cruz Biotechnology, Inc., Santa Cruz, CA, USA), rabbit anti-human EGF (Sigma Aldrich)) and anti-rabbit Alexa Fluor^®^ 488-conjugated secondary antibody (Invitrogen). For intracellular staining, the cells were prefixed with BD Cytofix/Cytoperm solution (BD Biosciences) at 4 °C for 20 min. The effect of **DSF-102** (2 µM, 50 µM) on AKT signaling was evaluated at different time points (15, 30, 60 min) using monoclonal *mouse*-anti-*human* phospho AKT (Ser473) (Proteintech Group, Inch, Rosemont, IL, USA] and Alexa Fluor^®^ 488-conjugated secondary antibody (Invitrogen). After co-administration with EGF (100 ng/mL), the inhibitory activity of **DSF-102** was studied investigating using FCM and the activation state of EGFR downstream targets after 30 and 60 min from stimulation. For this analysis, we used rabbit anti-human phospho(Thr202/Tyr204)-p44/42 ERK, rabbit anti-human phospho(Thr180-Tyr182)-p38 MAPK (both from Cell Signaling Technologies, Danvers, MA, USA), and anti-rabbit Alexa Fluor^®^ 488-conjugated secondary antibody (Invitrogen). FCM data were acquired with FACSCanto II flow cytometer (BD Biosciences) and expressed as mean fluorescence intensity (MFI) ± standard error of the mean (SEM). 

### 2.3. Computational Studies

Virtual screening, docking studies and molecular dynamics were run on a Linux Workstation (OS: Ubuntu 20.04; CPU: 32 core AMD Ryzen 9 3905X @ 3.5 GHx; GPU: Nvidia Quadro RTX 4000 8 GB).

#### 2.3.1. Virtual Screening and Docking Studies on DSF-102

The three-dimensional structures (“.mol2” format with explicit hydrogenation at pH = 7.2) of the compounds were prepared with OpenBabel ver. 3.1.1 starting from the SMILES code [31] and then converted in the appropriate “.pdbqt” files using AutoDock (AD) ver. 4.2 [32]. The structure of EGFR-ECD was retrieved from the Protein Data Bank (PDB-ID: 1YY9 [33]). The structure of cetuximab and the water molecules were removed. The apo-EGFR-ECD structure was converted to the appropriate “.pdbqt” file using AD ver. 4.2 [32].

The blind docking studies were conducted with QuickVina-W software [34]. The entire protein structure was contained in a box centered at *x* = 30.152, *y* = 2.064, *z* = −2.261 and of 84 × 90 × 100 Å dimension. For each ligand, up to 20 poses were generated with exhaustiveness = 100. The compounds were then ranked according to the score of the best pose. Docking of **DSF-102** was conducted using QuickVina-W software centering a box of 20 × 20 × 20 Å dimension on the binding site identified for isatin Schiff bases.

#### 2.3.2. Molecular Dynamics (MD) Simulations

All MD simulations were carried out using the Gromacs ver. 2021.1 [35,36]. The CHARMM36 forcefield [37] was used for both EGFR-ECD and **DSF-102**. The apo form of the protein and the complex were solvated with the TIP3P water model. Neutralization was performed using Na^+^ and Cl^−^ ions at a final concentration of 0.15 M. The PME method [38] was used to model the long-range electrostatic interactions, and the short-range Coulomb and van der Waals interactions were subjected to a cut-off of 1.2 nm. The bonds involving hydrogen atoms were constrained with the LINCS algorithm [39]. Rigid water molecules were constrained with the SETTLE algorithm. The input files for EGFR-ECD were prepared with the CHARMM-GUI web service [40,41] starting from the 1YY9 pdb structure, also retaining the glycan molecules. The input files for **DSF-102** were prepared through the CGenFF website (program version: 2.5; CGenFF version: 4.4) [42,43]. Both the apo EGFR-ECD and the complex **DSF-102**/EGFR-ECD were equilibrate with the NVT ensemble for 200 ps at T = 300 °K, using the velocity rescaling thermostat (0.1 ps), and dt = 0.001 fs [44]. Then, 200 ps of equilibration with the NPT ensemble were performed at T = 300 °K, P = 1 bar, and dt = 0.002 fs. The pressure was kept constant with the Berendsen barostat [45]. For the production runs, the NPT ensemble was used, replacing the Berendsen barostat with the Parrinello-Rahman [46] barostat and removing the positional restraints of the heavy atoms used in the equilibration steps. 

## 3. Results

### 3.1. Identification of Hit Compounds Targeting the EGFR-ECD

**Figure 2:**
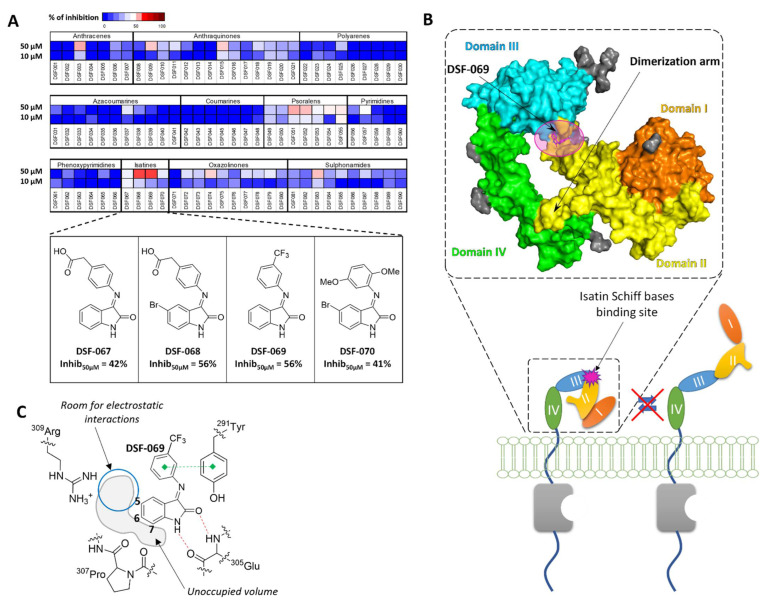
(**A**) Heat-map representing the percentage of EGFR dimerization inhibition at 50 and 10 µM. Structures and percentage of inhibition at 50 µM for the most interesting isatin Schiff bases are reported. (**B**) Top: binding site predicted for **DSF-069** (highlighted with magenta circles) on EGFR-ECD (surface representation). Glycans are depicted as gray spheres. Bottom: schematic representation of the binding site for isatin Schiff bases on EGFR-ECD and proposed mechanism of action, i.e., block of the receptor in the inactive/closed conformation. (**C**) Details of the interactions (docking simulation) between **DSF-069** and EGFR-ECD. Hydrogen bonds are depicted as red dashed lines; π-π interaction is depicted as green dashed line. The volume unoccupied by **DSF-069** is highlighted in gray. The possibility to functionalize the molecule with an electron-dense group is also shown.

The internal collection of compounds of the Pharmaceutical and Pharmacological Sciences Department of Padova University (about 8000 molecules) was initially submitted to virtual screening studies to identify potential EGFR-ECD binders. The conformation of inactive EGFR-ECD was extracted from the complex with cetuximab (PDB ID: 1YY9) [33]. Since there was no a priori information on the location of potential binding site(s) on the EGFR-ECD surface for small molecules, a blind docking approach was faced (i.e., the entire receptor surface was explored) using the QuickVina-W software [34]. The 90 top-scored compounds were screened in duplicate at two fixed concentrations (10 and 50 µM) for their ability to impair the EGFR dimerization using the PathHunter^®^ kit. Isatin Schiff-base derivatives constituted the most promising class of compounds, with **DSF-068** and **DSF-069** being the most potent inhibitors identified (Figure 2A).

### 3.2. Design, Synthesis, and SARs of Improved EGFR Inhibitors: Development of DSF-102

**Scheme 1:**
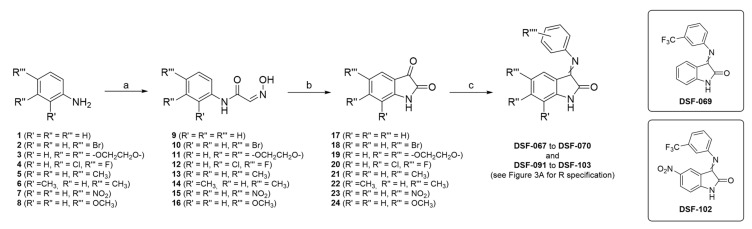
Synthesis of isatin Schiff-base derivatives. Reaction conditions: (**a**) chloral hydrate, NH_2_OH·HCl, Na_2_SO_4_, 1M HCl, 80 °C, 2 h; (**b**) conc. H_2_SO_4_, 60 °C, 15 min; (**c**) (4-aminophenyl)acetic acid or 3-aminobenzotrifluoride, AcOH, ethanol, reflux or MW irradiation. See Supplementary Materials for details. The structures of the initial hit (**DSF-069**) and of the most active compound in the series (**DSF-102**; vide post) are explicitly reported on the right.

The binding mode proposed by the virtual screening suggested that the isatin Schiff bases bound the protein at the interface between domains II and III (Figure 2B). In particular, (1) the indolinone scaffold formed two H-bonds with the amide backbone of ^305^Glu; (2) the phenylimino moiety interacted with the ^291^Tyr sidechain through a π-π stacking; (3) the phenylamino substituent formed one H-bond with the ^292^Glu amide-NH (in the case of **DSF-068** acetic acid function; not shown) or established non-polar interactions with the side chains of ^291^Tyr (in the case of **DSF-069** trifluoromethyl function). In accordance with the preliminary screening data (compare **DSF-067** and **DSF-068**), the docking simulation also suggested the possibility to functionalize the indolinone scaffold at 5, 6, or 7 positions, due to the presence of a small cleft formed by ^309^Arg and ^307^Pro (see the gray region in Figure 2C). Moreover, since the indolinone was suggested to point toward the sidechain of ^309^Arg, we also supposed that the introduction in position 5 of an electron-dense substituent can contribute to stabilize the binding through electrostatic interactions (see blue circle in Figure 2C). On these bases, to explore the possibility of further enhancing the inhibitory potency of the hit compounds, we synthesized and tested a focused series of analogues of compounds **DSF-067, −068** and **−069** (Scheme 1). Indeed, although **DSF-070 also** demonstrated promising activity, its remarkably lower solubility in aqueous media prompted us to abandon the 2,5-dimethoxyanilino derivatives. All the newly design derivatives were characterized by different groups (both in terms of bulkiness and electronic features) at positions 5, 6, and 7.

Briefly, isatin Schiff bases were synthesized starting from anilino derivatives **1**–**8** through a two-step strategy [22,47,48]: the amine was first condensed with chloral hydrate and hydroxylamine, and the amide intermediates **9**–**16** were then cyclized in concentrated sulfuric acid to yield the isatins **17**–**24**. Finally, the carbonyl function was condensed with either (4-aminophenyl)acetic acid or 3-aminobenzotrifluoride. The desired final compounds were obtained as a non-resolved mixture of *E* (major) and *Z* (minor) isomers: indeed, as already reported [49], the *E* and *Z* isomers are in equilibrium in solution. Newly synthesized compounds were then evaluated for their ability to impair the EGFR dimerization. 

**Figure 3:**
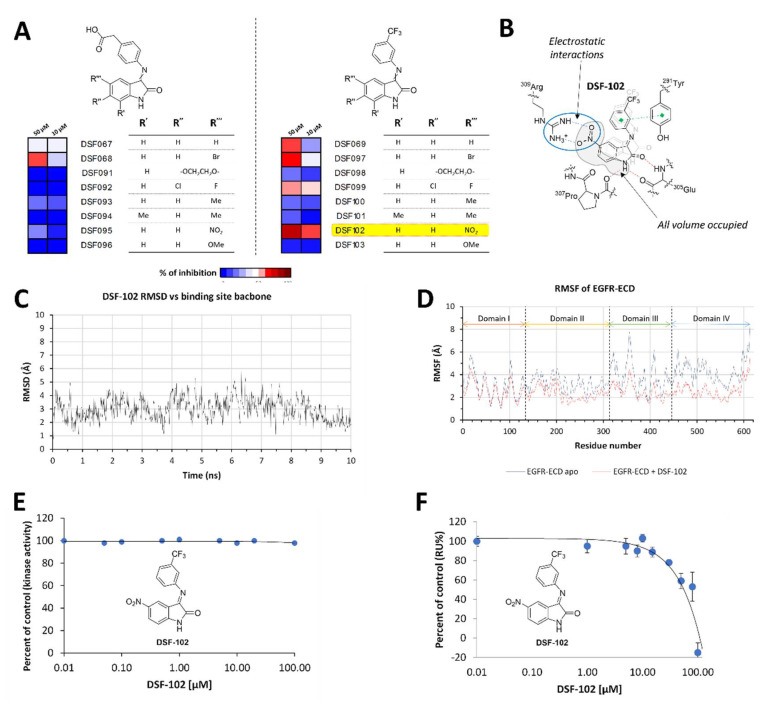
(**A**) Heat-map representing the percentage of EGFR dimerization inhibition at 50 and 10 µM. (**B**) Binding mode proposed for **DSF-102** and EGFR-ECD. Hydrogen bonds are depicted as red dashed lines; π-π interaction are depicted as green dashed lines; electrostatic interactions are depicted as blue dashed lines. The binding mode of **DSF-069** (light gray) is also reported to show how **DSF-102** resulted slightly shifted with respect to the parent compound, thus leading to a higher complementarity with the binding pocket. (**C**) Root mean squared deviation (RMSD, Å) for **DSF-102** during 10 ns of MD simulation. (**D**) Comparison of per-residue root mean squared fluctuation (RMSF, Å) obtained during 10 ns of MD simulation of apo EGFR-ECD (blue line) and **DSF-102** bound EGFR-ECD (red line). (**E**) Residual catalytic activity (%) of the isolated kinase domain of EGFR upon treatment with **DSF-102**. Please note that no inhibition was observed up to 100 µM. (**F**) **DSF-102** dependent inhibition of the binding between EGF and isolated EGFR-ECD as measured by SPR analysis.

The results from the biological evaluation (Figure 3A) revealed that the *p*-acetic acid derivatives barely tolerated the further functionalization at the indolinone ring, with the only exception being 5-Br (**DSF-068**). Conversely, the corresponding *m*-CF_3_ compounds were generally more active, with compound **DSF-102** being the most active in the series. More in detail, the introduction of an electron-withdrawing group (either halide or nitro) at position 5 led to compounds more active than the starting hit (compare **DSF-069** with both **DSF-097**, **DSF-099,** and **DSF-102**), while the introduction of a methyl function led to a marked reduction in activity (compare **DSF-069** with **DSF-100**). These data agreed with the assumption that an electron-dense group at position 5 (bromine in **DSF-097**, fluorine in **DSF-099** and nitro in **DSF-102**) would have established electrostatic interactions with the ^309^Arg sidechain, whereas apolar functions would have impaired the interaction. The further introduction of a substituent at position 7 was even more detrimental (compare the 5-methyl **DSF-100** with the 5,7-dimethyl **DSF-101**). Finally, the introduction of bulky group at position 5 (see compounds **DSF-098** and **DSF-103**) led to almost inactive compounds. 

Since the biological evaluation of the newly synthesized compounds agreed with the predicted binding site for isatin Schiff bases (i.e., a region between domains II and III of EGFR-ECD; Figure 2B), additional molecular docking simulations were conducted with **DSF-102**. These studies further confirmed that the nitro function established electrostatic interactions with the polar head of ^309^Arg (Figure 3B). We also noted that the docking pose of **DSF-102** did not perfectly overlap with that predicted for **DSF-069**. Indeed, the strong interaction between the nitro and the arginine caused a slight relocation of the compound that resulted able to occupy almost all the binding site (Figure 3B). The amide-NH of **DSF-102** was found to be almost equidistant from the amide C=O of both ^305^Glu and ^307^Pro, whereas the interactions between the indolinone C=O with the amide-NH of ^305^Glu as well as the π-π stacking between the phenylamino moiety and the ^291^Tyr sidechain (already observed for **DSF-069**) were retained. Taken together, these data explained the highest activity for **DSF-102** in the series. This binding mode also justified the very low activity of **DSF-094** and **DSF-101**: the ^307^Pro sidechain, indeed, did not allow the functionalization of the isatin derivatives at position 7. Similarly, bulky substituents at positions 6 and/or 7 (see compounds **DSF-091**, **−092**, **−098** and **−099**) led to inactive or poorly active analogues since the ^309^Arg sidechain limited the volume of the binding site. Molecular dynamics (MD) simulations (10 ns) were also run on both apo EGFR-ECD and **DSF-102** bound EGFR-ECD to further confirm the reliability of the proposed binding mode. Considering the high protein flexibility (see Figure S6A for RMSD of both apo EGFR-ECD and **DSF-102**-bound EGFR-ECD), **DSF-102** remained almost stably bound to the EGFR-ECD during the simulation time (Figure 3C). An average number of 1.85 ± 0.53 H-bonds between **DSF-102** and the protein were established during the simulation (Figure S6B). Distance analysis showed that the H-bond between the C=O function of **DSF-102** and the amide-NH of ^305^Glu was retained during the MD simulation (average distance: 1.97 ± 0.22 Å; see Figure S6C), whereas the amide-NH of **DSF-102** established a weaker and less stable interaction with the amide-C=O of ^305^Glu (average distance: 2.59 ± 0.51 Å; see Figure S6D). However, after the first 4 ns of simulation, **DSF-102** started to establish a H-bond with the amide-C=O of ^307^Pro (average distance_0–4 ns_: 2.41 ± 0.64 Å; average distance_4–10 ns_: 2.01 ± 0.33 Å see Figure S6E) that compensated the weakness of the interaction with the amide-C=O of ^305^Glu. The π-π interaction between the trifluoromethylphenyl function and the ^291^Tyr sidechain as well as the electrostatic interaction between the nitro function of **DSF-102** and the ^309^Arg sidechain were retained during the entire simulation. The binding with **DSF-102** led to the reduction in the flexibility of EGFR-ECD: indeed, the comparison of the per-residue backbone root mean squared fluctuation (RMSF) of apo- (Figure 3D, blue line) with the liganded- (Figure 3D, red line) EGFR-ECD clearly showed that domains II-IV were stabilized by **DSF-102**, while no effects were observed on domain I.

Dose-response curves (inhibition of EGFR dimerization measured using the PathHunter assay) were then determined for the most representative compounds, i.e., **DSF-097** (IC_50_ = 38.8 ± 3.6 µM), **DSF-099** (IC_50_ = 27.7 ± 4.5 µM) and **DSF-102** (IC_50_ = 13.2 ± 2.1 µM), further highlighting compound **DSF-102** as the most active one. Interestingly, **DSF-102** also showed promising computed drug-like properties [50] (see Figure S2 in Supplementary Materials for calculated values). To exclude that **DSF-102** acted by interacting with the EGFR-TKD, its dissociation constants with the ATP-binding domain (K_dEGFR-TKD_) was also measured. Indeed, the PathHunter screening assay measured the reduction of the receptor activity that can either arise from the impairment of the EGF-mediated dimerization (in the case of EGFR-ECD binders) or from the inhibition of the kinase activity (for EGFR-TKD binders). The compound, tested by the KinomeScan assay service of DiscoverX, resulted unable to inhibit the isolated EGFR-TKD (K_dEGFR-TKD_ > 100 µM; Figure 3E), again confirming that the EGFR-ECD was the effective target of our compounds. To further investigate this hypothesis, Surface Plasmonic Resonance (SPR) experiments were conducted. First, purified EGFR-ECD was immobilized on a CM5 sensor chip, and solutions of EGF (0 to 1 mM) were subsequently injected at increased concentrations. SPR resolved the interaction and provided a dissociation constant, K_d_, of 158 ± 16 nM (Figure S1) that agreed with literature data [51]. Next, a competition experiment was performed to assess whether the presence of **DSF-102** would reduce the binding of EGF to EGFR. A solution of EGF was incubated with different concentrations of **DSF-102**, and the resulting mixture was flowed over the same EGFR-coated sensor chip. A significant and dose-dependent decrease of EGF binding was observed, confirming that the presence of the hit compound reduces the interaction of EGF to its receptor (Figure 3F).

### 3.3. Effect of DSF-102 on EGFR Signaling and Trafficking

**Figure 4:**
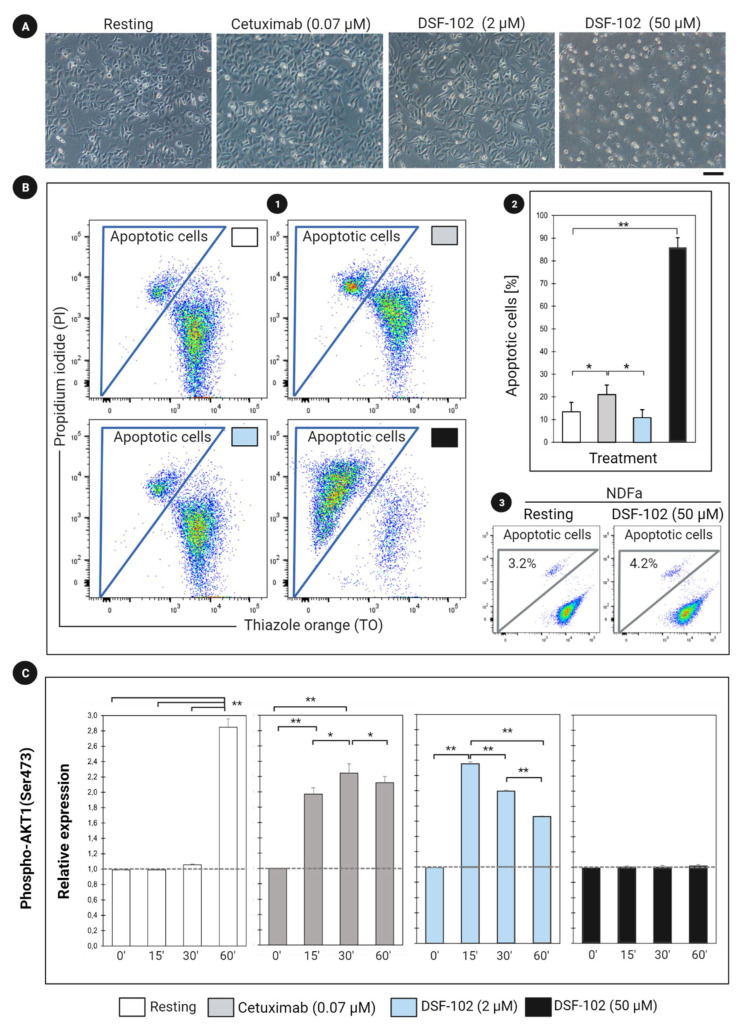
Study of the biological activity of **DSF-102** (2 µM or 50 µM) on A549 cell line. Assessment of cell death by (**A**) optical microscopy analysis (scale bar: 25 µm) and (**B**) apoptosis analysis by flow cytometry after 24 h of stimulation. A549 cells kept under resting conditions or treated with cetuximab (0.07 µM) (B1, B2), or primary dermal fibroblasts (NDFa) (B3) were used as controls. Percentage of apoptotic cells were discriminated using TO/PI assay according to the manufacturer’s instructions. (**C**) Evaluation of phospho AKT 1(Ser473) expression by flow cytometry at different time points (15, 30, 60 min) from stimulation with **DSF-102** (2 µM or 50 µM). Resting cells and samples treated with cetuximab (10 µg/mL; 0.07 µM) were used as reference. Flow cytometry data were expressed as MFI ± SEM. * (*p* ≤ 0.05); ** (*p* ≤ 0.01).

Based on IC_50_ values (EGFR dimerization inhibition), the compound **DSF-102** was selected for further biological evaluations on adenocarcinoma A549 cell line that is known to overexpress EGFR and to show KRAS mutations in codon 12 [21]. In the present study, **DSF-102** caused in vitro a marked dose-dependent decrease in cell viability after 24 h of treatment. As assessed by optical microscopy analysis (Figure 4A) and TO/PI test (Figure 4B), **DSF-102** at higher concentration (50 µM) induced apoptosis in 86% of cells (*p* ≤ 0.01) after 24 h of treatment, compared to control (untreated) samples (13.4%). In parallel, the exposure to cetuximab (10 µg/mL; 0.07 µM) promoted only a limited growth inhibitory effect (Figure 4A), leading to 21% of apoptotic cells (*p* ≤ 0.05; Figure 4B), in accordance with literature data [30,52]. It is of note that when primary normal dermal fibroblasts (NDFa) were treated for 24 h with **DSF-102** at 50 µM concentration, no significative reduction on cell viability was observed (Figure 4B3), suggesting that the cytotoxic effects observed in A549 were strongly related to EGFR inhibition and that **DSF-120** might have negligible off-target effects even at high concentration and with prolonged treatment. Based on these observations and considering the short time of treatment required for investigating the downstream effects of the EGFR inhibition as well as its internalization, we decided to evaluate the effects of **DSF-102** using a high concentration (i.e., 50 µM), to achieve significative differences between treated and untreated samples. When the biological effects of **DSF-102** were evaluated on EGFR-dependent signal cascade (Figure 4C), a higher time- and concentration-dependent reduction in phosphorylation of Akt1 was observed in comparison to cetuximab, suggesting that **DSF-102** (50 µM) overcame the anti-EGFR drug resistance already reported in A549 cells [21], and interfered with aberrant RAS signaling [52]. Serum stimulation is known to cause brief peaks of AKT phosphorylation followed by a moderate steady-state. In our experiments, the samples were cultured in medium added with 2% FBS. In resting cells, the peak of AKT1 phosphorylation at Ser473 was observed at 60 min, while at previous time points a moderate steady-state was detected. Modeling studies assume this peak and decline behavior of AKT phosphorylation as dependent on canonical/not canonical AKT activation or receptor internalization [53]. The treatment with cetuximab or **DSF-102** (50 µM) promoted AKT phosphorylation from 15 to 60 min with a peak at 30 min and 15 min, suggesting that AKT dynamics are not inhibited but only modulated under these experimental conditions. In contrast, **DSF-102** demonstrated a strong inhibitory effect on AKT signaling when used at 50 µM.

**Figure 5:**
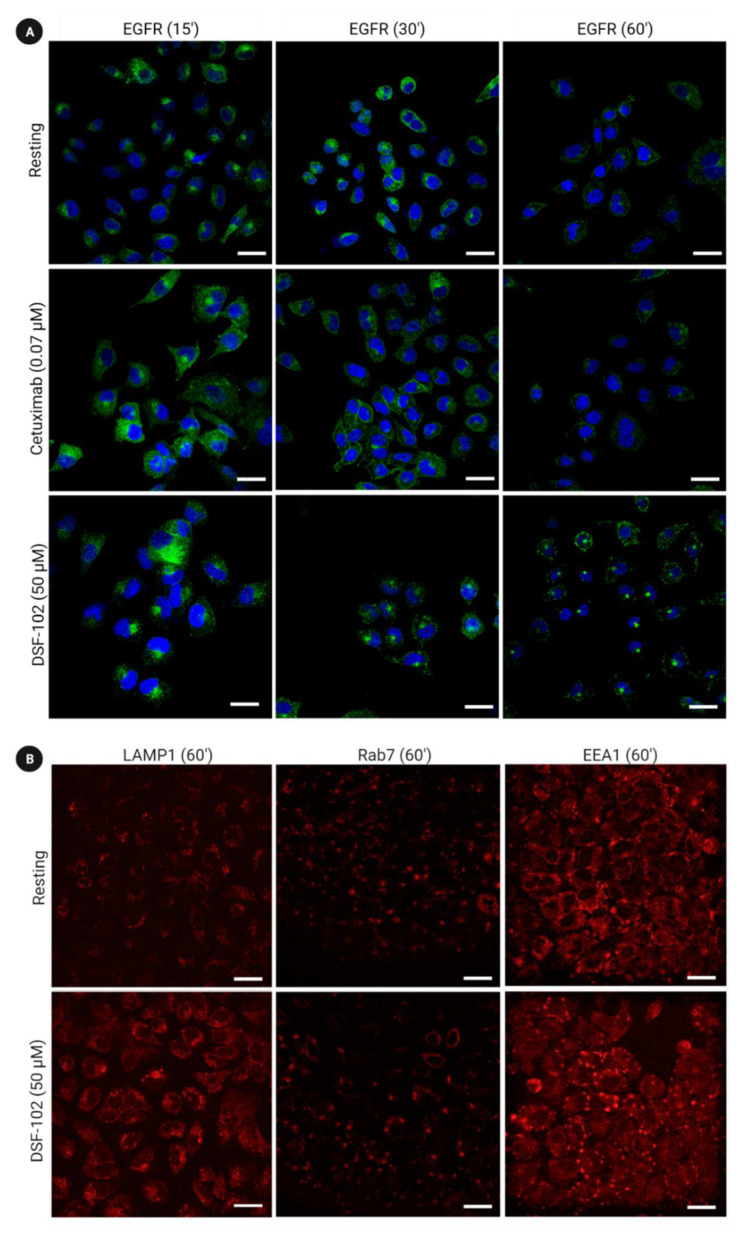
(**A**) Confocal microscopy images showing the trafficking kinetics of membrane EGFR pH (green) under resting conditions and after treatment (from 15 to 60 min) with **DSF-102** (50 µM) or cetuximab. Cellular nuclei were counterstained with DAPI (blue). Scale bar: 10 µm. (**B**) Immunofluorescence analysis of EEA1, Rab7 and LAMP1 (red) in fixed and permeabilized cultures compared to resting cells, at 60 min after treatment with **DSF-102** (50 µM). Scale bar: 10 µm.

To control downstream signaling, EGF-bound EGFR is known to internalize by coated pits and to be sorted in early endosomes for recycling or degradation [9,54]. After synthesis and delivery to the cell surface, EGFR is turned over with half-life ranging from 8 to 24 h, depending on the cell type, the level of EGFR expression, and activation by EGF [55]. In this study, the detection of EGFR from 15′ to 60′ by immunofluorescence demonstrated plasma membrane recycling of EGFR in resting cells and in samples treated with cetuximab (Figure 5A). Conversely, after the administration of **DSF-102** (50 µM)**,** A549 cells showed EGFR accumulation on the membrane and in the perinuclear region (Figure 5A) from early to later time of stimulation. Compared to control samples, **DSF-102** (50 µM) promoted the formation of numerous clustered vesicles expressing EEA1 [56] (Early Endosome Antigen 1) and increased the expression of LAMP1 [57] (Lysosomal-Associated Membrane Protein 1; Figure 5B). In parallel, we ascertained the expression of Rab7 [58] (Ras-related protein Rab-7) that is essential for the degradation of signaling receptors, being responsible for sorting them into late endosome and of their transport to lysosomes (Figure 5B). 

**Figure 6:**
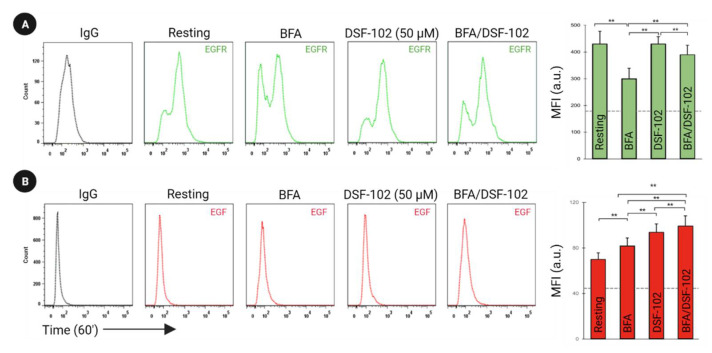
Flow cytometry analysis of (**A**) membrane EGFR (green line) and (**B**) intracellular EGF (red line) at 60 min of stimulation with 50 µM **DSF-102** alone or combined with brefeldin A (BFA/**DSF-102**). Resting cells and BFA-treated samples were used as controls. To detect only intracellular EGF, flow cytometry was coupled with acid washing. Data were expressed as MFI ± SEM. ** (*p* ≤ 0.01).

To investigate the mechanism of action of **DSF-102**, the membrane EGFR content and the intracellular EGF expression were measured by flow cytometry at 60 min from stimulation. To discriminate EGFR internalized or stabilized from the receptor de novo synthetized and exposed on membrane, the protein trafficking inhibitor brefeldin A (BFA) was used [59]. As shown in Figure 6A, brefeldin A (BFA) is a known inhibitor of early biosynthetic trafficking (RIF) from the endoplasmic reticulum to the Golgi apparatus. Bryant et al. [60] demonstrated that the treatment with BFA inhibits the biosynthetic trafficking of EGFR to the plasma membrane (PM). In our study, the inhibition by BFA has been performed to better monitor the change in the expression level of (i) EGFR on plasma membrane (PM); and (ii) cytoplasmic EGF, following stimulation with EGF, cetuximab or **DSF-102**. At 60′ from BFA treatment, the biosynthetic trafficking to PM demonstrated to be negatively regulated. After stimulation with **DSF-102** alone or concomitantly administered with BFA, the increased expression of EGF was assumed to be a compensatory effect against the block of EGFR signaling. In accordance with the hypothesis that **DSF-102** could act as a membrane inhibitor of EGFR trafficking, we detected a higher expression of pEGFR in samples co-treated with BFA and **DSF-102**. Taken together, these data suggested that **DSF-102** stabilized the EGFR-ECD in a monomeric inactive state on the membrane, thus increasing the turnover rate of EGFR to restore the basal signaling. The treatment with **DSF-102** (50 µM) alone or in combination with BFA (Figure 6B) caused progressive increasing amounts of EGF, further supporting the proposed mechanism of action. 

**Figure 7:**
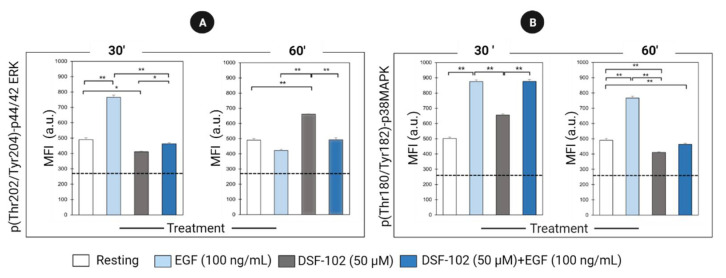
Flow cytometry analysis of DSF-102 activity on EGFR signaling. (**A**) p44/42 ERK and (**B**) p38 MAPK activation at 30 and 60 min following the co-administration of **DSF-102** (50 µM) and EGF (100 ng/mL). Samples treated with only EGF or **DSF-102**, and resting cells were used as controls. Data were expressed as MFI ± SEM. * (*p* ≤ 0.05); ** (*p* ≤ 0.01).

**Figure 8:**
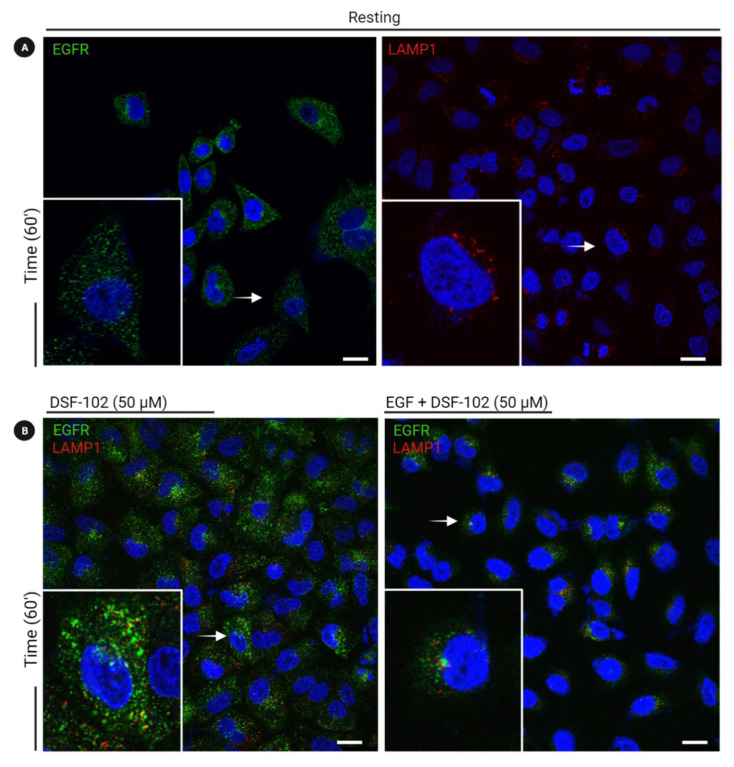
Confocal microscopy evaluation. Images show the localization of EGFR (green) and lysosomal marker LAMP1 (red) in A549 cells resting (time 0′) (**A**) or treated for 60 min with **DSF-102** (50 µM) and/or EGF (100 ng/mL) (**B**). For each picture, a region of interest of was included at higher magnification. Nuclei were counterstained with DAPI (blue). Scale bar: 10 µm.

The inhibitory activity of **DSF-102** was furtherly investigated evaluating the p44/42 ERK and p38 MAPK activation induced by EGF exogenously administered. Our data showed that **DSF-102** negatively modulated ERK signaling at 30 min from stimulation (Figure 7A) while at 60 min reversed the effect of exogenous EGF on both pathways (Figure 7A,B) and promoted the lysosomal degradation of EGFR (Figure 8A,B).

The cytotoxic effects of **DSF-102** against human triple negative breast MDA-MB-231 and human colon HCT-15 cancer cells, overexpressing EGFR, as well as on human melanoma cells, poorly expressing EGFR, were also determined, to obtain preliminary data on the specificity of action of the compound (Table 1). 

Remarkably, **DSF-102** was ineffective against human melanoma cells (IC_50_ > 100 µM), whereas it showed a prominent in vitro antiproliferative activity against human EGFR-overexpressing cells. In particular, **DSF-102** elicited and IC_50_ value in the low micromolar range (12.8 µM) against human colon HCT-15 cancer cells, which are characterized by EGFR overexpression and KRAS mutation at codon 13. 

These data clearly suggest that a correlation between the EGFR expression level and the cytotoxic efficacy of **DSF-102** can be outlined.

## 4. Discussion

The EGFR is a well-known oncogene involved in the onset and progression of different types of cancer. To date, the pharmacological inhibition of EGFR is accomplished either by blocking the receptor activity (i.e., using monoclonal antibodies targeting the extracellular domain) or by impairing its kinase activity (i.e., using ATP-mimic small molecules).

In this work, we employed virtual screening and medium throughput screening techniques to identify a new class of small molecules (namely isatin Schiff bases) able to impair the EGFR functions by targeting the extracellular domain of EGFR. Our preliminary medicinal chemistry efforts further allowed the development of a promising compound (named **DSF-102**) with improved activity with respect to the initial hit. The binding to EGFR-ECD was demonstrated through SPR experiments, which showed that **DSF-102** was able to reduce the interaction between EGFR and EGF. The absence of ATP-mimic properties further confirmed that **DSF-102** exerted its inhibitory activity on EGFR by targeting the extracellular domain of the receptor. The binding mode of **DSF-102** with EGFR-ECD was investigated by means of molecular docking and molecular dynamics simulations. The computational experiments suggested that the molecule interacted with the protein at the interface between domains II and III, probably leading to partial stabilization of the receptor in the closed (inactive) conformation. **DSF-102** induced cytotoxic effects in the EGFR-overexpressing cells A549 and altered the downstream signaling. **DSF-102** induced a higher production of EGF and EGFR, but also a simultaneous increase in receptor internalization, lysosomal accumulation, and degradation. Moreover, **DSF-102** was demonstrated to negatively control EGFR signaling in A549 cells. **DSF-102** showed different cytotoxic effects on human cancer lines: the viability of low expressing EGFR cells (i.e., A375) was not affected by up to 100 µM compound concentration. Conversely, high expressing EGFR cells (i.e., MDA-MB-231 and HCT-15) were sensitive to **DSF-102**.

## 5. Conclusions

Our data suggest that **DSF-102** can be considered to be a promising hit compound for the further development of extracellular EGFR inhibitors bearing the isatin Schiff-base scaffold. The effects elicited by **DSF-102** have not been confronted in terms of binding mode, binding site, or induction of EGFR-ECD structural rearrangement with those caused by cetuximab. Indeed, the comparison between a small molecule and a monoclonal antibody cannot be properly made at this molecular level, even though both **DSF-102** and cetuximab share the same target (i.e., EGFR-ECD) and the same initial events (i.e., impairment of receptor dimerization and autophosphorylation). Instead, the herein reported comparison of the different effects between **DSF-102** and cetuximab on MAPK phosphorylation and induction of apoptosis in A549 cells suggested that KRAS-mediated resistance could also be overcome by inducing a different pathway of EGFR recycling and degradation in addition to using drug combinations as previously reported [61]

At the molecular level, the mechanism of action proposed for **DSF-102** consisted of the (partial) stabilization of the closed/inactive form of EGFR-ECD (as suggested by molecular docking and molecular dynamics simulations), thus preventing the binding with EGF (as demonstrated by SPR studies that showed a reduction in EGFR-ECD/EGF interaction) and blocking the receptor dimerization and autophosphorylation (as demonstrated by the experiments with the PathHunter kit). The presence of a “useless” EGFR on the cell surface was then supposed to induce a higher receptor internalization.

To the best of our knowledge, only two small molecules (**DSF-102** and **DPBA** [20]) have been so far identified that block the EGFR activity by binding the extracellular domain and without acting as ATP-mimic compounds. Of note, both compounds show on-target activity in the micromolar range (Kd ≃ 40 µM for **DPBA**; IC_50-dimerization_ ≃ 13 µM for **DSF-102**), which are promising values for these hit compounds, considering that the EGFR-ECD has not evolved to bind small molecules. Accordingly, **DSF-102** deserves additional medicinal chemistry studies for improving its potency with the aim to develop a new pharmacological strategy for the treatment of EGFR-dependent diseases.

## Figures and Tables

**Table 1:** Cytotoxicity data for DSF-102 against different cell lines. Cells (5–8 × 10^3^ mL^−1^) were treated for 24 h with increasing concentrations of the tested compound. The cytotoxicity was assessed by the MTT test. IC_50_ values were calculated by a four-parameter logistic model (*p* < 0.05). S. D. = standard deviation.

	IC_50_ (µM) ± S. D.		
	**A375**	**MDA-MB-231**	**HCT-15**
**DSF-102**	>100	41.3 ± 6.7	12.8 ± 2.1

## Data Availability

The data presented in this study are available in this article (and Supplementary Materials).

## Supplementary Materials

The following supporting information can be downloaded at: https://www.mdpi.com/article/10.3390/cancers14153647/s1, synthesis and characterization of isatin Schiff bases; surface plasmonic resonance (SPR) experiments; Figure S1: binding of EGF to EGFR monitored by surface plasmonic resonance; Figure S2: drug likeness properties of **DSF-102**; Figure S3: HPLC analysis of **DSF-102**; Figure S4: 1H-NMR of **DSF-102**; Figure S5: 13C-NMR of **DSF-102**; Figure S6: results from MD studies; Table S1: list of screened compounds, SMILES code and % of inhibition at 50 and 10 µM [22,47,48,50,62].
