# Supplementary material for: Preliminary Discovery of Small Molecule Inhibitors of Epidermal Growth Factor Receptor (EGFR) That Bind to the Extracellular Domain
Source: Cancers (Basel). 2022 Jul 27;14(15):3647. doi: 10.3390/cancers14153647 (PMC9367601; doi:10.3390/cancers14153647)
Supplement: Supplementary file 1 [file cancers-14-03647-s001.zip › cancers-1801327-supplementary.pdf]

SUPPLEMENTARY MATERIALS

Preliminary discovery of small molecule inhibitors of Epidermal Growth Factor Receptor (EGFR) that bind to the extracellular domain

Rosa Di Liddo [1], Marco Verona [1], Christian Vaccarin [1,2], Laura Acquasaliente [1], Sandra Schrenk [1], Monica Piccione [1], Carola Cenzi [1], Michele De Franco [1], Matteo Dal Prà[1], Giovanni Ribaudo [3], Maria Grazia Ferlin [1,†], Maria Teresa Conconi [1], Adriana Chilin [1], Valentina Gandin [1] and Giovanni Marzaro [1,*]

[1] Department of Pharmaceutical and Pharmacological Sciences, University of Padova, Via Marzolo 5, I-35131 Padova, Italy; rosa.diliddo@unipd.it (R.D.L.); marco.verona@phd.unipd.it (M.V.); christian.vaccarin@phd.unipd.it (C.V.); laura.acquasaliente@unipd.it (L.A.); sandra89.schrenk@gmail.com (S.S.); monica.piccione@studenti.unipd.it (M.P.); carola.cenzi@iov.veneto.it (C.C.); miky_defra@hotmail.com (M.D.F.); matteo.dal-pra@autifony.com (M.D.P.); mariagrazia.ferlin@unipd.it (M.G.F.); mariateresa.conconi@unipd.it (M.T.C.); adriana.chilin@unipd.it (A.C.); valentina.gandin@unipd.it (V.G.)

[2] Center for Radiopharmaceutical Sciences, ETH-PSI-USZ, Paul Scherrer Institute, 5232 Villigen-PSI, Switzerland

[3] Department of Molecular and Translational Medicine, University of Brescia, Viale Europa 11, I-25123 Brescia, Italy; giovanni.ribaudo@unibs.it

* Correspondence: Correspondence: giovanni.marzaro@unipd.it; Tel.: +39-049-827-5024

† Retired.

Experimental details:

1. Synthesis and characterization of isatin Schiff bases........page S2
2. Surface Plasmonic Resonance (SPR) experiments........page S7
   2.1. EGFR-EGF interaction........page S7
   2.2. **DSF-102** competition experiments........page S8

Supplementary Table and Figures:

Figure S1. Binding of EGF to EGFR monitored by surface plasmonic resonance........page S8
Figure S2. Drug likeness properties of **DSF-102**........page S9
Figure S3. HPLC analysis of **DSF-102**........page S9
Figure S4. 1H-NMR of **DSF-102**........page S10
Figure S5. 13C-NMR of **DSF-102**........page S10
Figure S6. Results from MD studies........page S11
Table S1. List of screened compounds, SMILES code and % of inhibition at 50 and 10 μM........page S12

Supplementary Bibliography........page S15

1. Synthesis and characterization of isatin Schiff bases

All solvent and starting materials were purchased from commercial suppliers (Sigma Aldrich and Carlo Erba) and used without further purifications. Thin layer chromatography (TLC) was performed on pre-coated plates of silica gel 60 with fluorescent indicator UV254 (0.2 mm, Merck); column chromatography was done with silica gel 60 (0.063-0.100 mm, Merck). The microwave-assisted synthetic procedures were conducted on a CEM Discover monomode reactor with the temperature monitored by a built-in infrared sensor and the automatic control of the power; all the reactions were performed in closed devices with pressure control. The ^{1}H- and ^{13}C-NMR spectra were recorded at room temperature on a Bruker 300 spectrometer; chemical shifts are reported in ppm and are referred to residual solvent peaks. Coupling constants (J) are given in Hz. The ratio E/Z was determined on the basis of 1H-NMR peak areas. The purity of the compounds (higher than 99.5% for all the compounds) was checked by HPLC. HRMS spectra were obtained using a Applied Biosystem Mariner system 5220 with direct injection of the sample.

Compounds were synthesized according to the following general scheme:

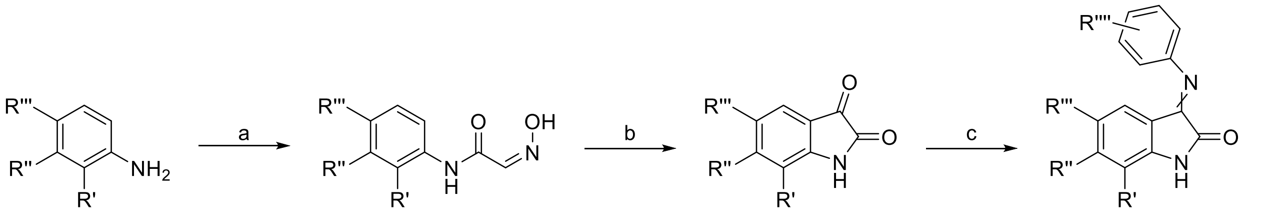

1 (R' = R'' = R''' = H)
2 (R' = R'' = H, R''' = Br)
3 (R' = H, R'' = R''' = -OCH_2CH_2O-)
4 (R' = H, R'' = Cl, R''' = F)
5 (R' = R'' = H, R''' = CH_3)
6 (R' =CH_3, R'' = H, R''' = CH_3)
7 (R' = R'' = H, R''' = NO_2)
8 (R' = R'' = H, R''' = OCH_3)

9 (R' = R'' = R''' = H)
10 (R' = R'' = H, R''' = Br)
11 (R' = H, R'' = R''' = -OCH_2CH_2O-)
12 (R' = H, R'' = Cl, R''' = F)
13 (R' = R'' = H, R''' = CH_3)
14 (R' =CH_3, R'' = H, R''' = CH_3)
15 (R' = R'' = H, R''' = NO_2)
16 (R' = R'' = H, R''' = OCH_3)

17 (R' = R'' = R''' = H)
18 (R' = R'' = H, R''' = Br)
19 (R' = H, R'' = R''' = -OCH_2CH_2O-)
20 (R' = H, R'' = Cl, R''' = F)
21 (R' = R'' = H, R''' = CH_3)
22 (R' =CH_3, R'' = H, R''' = CH_3)
23 (R' = R'' = H, R''' = NO_2)
24 (R' = R'' = H, R''' = OCH_3)

DSF-067 to **DSF-069**
and
DSF-091 to **DSF-103**
(see teble below for R specification)

Reaction conditions: a) chloral hydrate, $NH_2OH{\cdot}HCl$, Na_2SO_4, 1M HCl, 80°C, 2h; b) conc. H_2SO_4, 60°C, 15 min; c) (4-aminophenyl)acetic acid or 3-aminobenzotrifluoride, AcOH, ethanol, reflux or MW irradiation.

Compounds **9-24** were synthesized according to literature methods[1,2,3]

Compounds **DSF-067/-069** and **DSF-091/-103** are herein reported in detail.

ID	Starting isatin	R′	R′′	R′′′	R′′′′
**DSF-067**	**17**	H	H	H	*p*-CH_2COOH
**DSF-068**	**18**	H	H	Br	*p*-CH_2COOH
**DSF-069**	**17**	H	H	H	*m*-CF_3
**DSF-091**	**19**	H	-OCH_2CH_2O-		*p*-CH_2COOH
**DSF-092**	**20**	H	Cl	F	*p*-CH_2COOH
**DSF-093**	**21**	H	H	Me	*p*-CH_2COOH
**DSF-094**	**22**	Me	H	Me	*p*-CH_2COOH
**DSF-095**	**23**	H	H	NO_2	*p*-CH_2COOH
**DSF-096**	**24**	H	H	OMe	*p*-CH_2COOH
**DSF-097**	**18**	H	H	Br	*m*-CF_3
**DSF-098**	**19**	H	-OCH_2CH_2O-		*m*-CF_3
**DSF-099**	**20**	H	Cl	F	*m*-CF_3
**DSF-100**	**21**	H	H	Me	*m*-CF_3
**DSF-101**	**22**	Me	H	Me	*m*-CF_3
**DSF-102**	**23**	H	H	NO_2	*m*-CF_3
**DSF-103**	**24**	H	H	OMe	*m*-CF_3

General Procedure for the Synthesis of Isatin Schiff-bases. In a round-bottomed flask, isatin derivative **17-24** (1.0 mmol) was dissolved in hot absolute ethanol (3 mL), and then an equimolar amount of suitable substituted aniline was added. The mixture is acidified with glacial acetic acid (100 μL) and refluxed overnight. The obtained precipitate was filtered, washed with ethanol and re-crystallised from ethanol. In some cases, the reaction was performed under microwave irradiation at 200W, 115 °C for 30 minutes, dispersing equimolar amounts of reagents (1 mmol each) in 3 mL of ethanol and adding 100 μL of glacial acetic acid.

(E,Z)-2-(4-(2-oxoindolin-3-ylideneamino)phenyl)acetic acid (**DSF-067**). Compound **DSF-067** was prepared by reacting compound **17** with 2-(4-aminophenyl)acetic acid under conventional heating. Yield 34%.
Ratio E/Z: 85:15; mp = 227-229°C; IR (KBr): ṽ (cm^{-1}) = 3244 (NH), 1719 (C=O), 1201 (C=O); HRMS (ESI-TOF) for $C_{16}H_{13}N_2O_3$ $(M+H)^+$: calcd = 281.0921, found = 281.1003
Isomer E: ^1H NMR (300 MHz, DMSO-d_6) δ 12.34 (broad s, 1H), 10.96 (broad s, 1H), 7.37-7.32 (m, 3H), 6.95-6.92 (m, 2H), 6.90 (dd, J=7.8, J=0.8, 1H), 6.73 (td, J=7.8, J=0.8, 1H), 6.45 (dd, J=7.8, J=0.8, 1H), 3.64 (s, 2H); ^{13}C NMR (75 MHz, DMSO-d_6) δ 173.2, 164.0, 155.4, 149.4, 147.4, 134.9, 132.3, 131.0, 125.8, 122.1, 117.8, 116.2, 112.0, 40.6
Isomer Z: ^1H NMR (300 MHz, DMSO-d_6) δ 12.34 (broad s, 1H), 10.85 (broad s, 1H), 7.58 (dd, J=7.5, J=0.9, 1H), 7.47 (td, J=7.5, J=0.9, 1H), 7.22-7.18 (m, 2H), 7.06 (td, J=7.5, J=0.9, 1H), 7.00-9.62 (m, 2H), 6.86 (dd, J=7.5, J=0.9, 1H), 3.56 (s, 2H); ^{13}C NMR (75 MHz, DMSO-d_6) δ 172.8, 158.9, 153.2, 147.8, 146.0, 134.6, 131.8, 129.7, 123.2, 122.8, 122.0, 119.9, 111.2, 40.6

(E,Z)-2-(4-(5-bromo-2-oxoindolin-3-ylideneamino)phenyl)acetic acid (**DSF-068**). Compound **DSF-068** was prepared by reacting compound **18** with 2-(4-aminophenyl)acetic acid under conventional heating. Yield 31%.
Ratio E/Z: 81:19; mp = 235-237°C; IR (KBr): ṽ (cm^{-1}) = 3231 (NH), 1726 (C=O), 1232 (C=O); HRMS (ESI-TOF) for $C_{16}H_{12}BrN_2O_3$ $(M+H)^+$: calcd = 359.0026, found = 359.0018
Isomer E: ^1H NMR (300 MHz, DMSO-d_6) δ 12.35 (broad s, 1H), 11.12 (broad s, 1H), 7.53 (dd, J=8.4, J=2.0, 1H), 7.40-7.36 (m, 2H), 6.98-6.94 (m, 2H), 6.88 (d, J=8.4, 1H), 6.50 (d, J=2.0, 1H), 3.65 (s, 2H); ^{13}C NMR (75 MHz, DMSO-d_6) δ 173.2, 163.5, 158.5, 154.4, 149.0, 146.5, 137.0, 131.1, 128.0, 117.8, 114.0, 113.4, 40.6
Isomer Z: ^1H NMR (300 MHz, DMSO-d_6) δ 12.35 (broad s, 1H), 11.00 (broad s, 1H), 7.69 (d, J=2.0, 1H), 7.62 (dd, J=8.3, J=2.0, 1H), 7.23-7.20 (m, 2H), 7.04-7.01 (m, 2H), 6.83 (d, J=8.3, 1H), 3.57 (s, 2H); ^{13}C NMR (75 MHz, DMSO-d_6) δ 172.5, 163.6, 159.0, 152.3, 147.2, 145.0, 136.6, 129.7, 125.5, 120.2, 117.8, 114.4, 113.3, 40.6

(E,Z)-3-(3′-(trifluoromethyl)phenylimino)indolin-2-one (**DSF-069**). Compound **DSF-069** was prepared by reacting compound **17** with 3-(trifluoromethyl)aniline under conventional heating. Yield 27%.
Ratio E/Z: 69:31; mp = 251-254°C; IR (KBr): ṽ (cm^{-1}) = 3424 (NH), 1713 (C=O), 1330 (C-F); HRMS (ESI-TOF) for $C_{15}H_{10}F_3N_2O$ $(M+H)^+$: calcd = 291.0740, found = 291.0728
Isomer E: ^1H NMR (300 MHz, DMSO-d_6) δ 11.01 (broad s, 1H), 7.70 (t, J=7.2, 1H), 7.62-7.58 (m, 1H), 7.39-7.33 (m, 3H), 6.92 (d, J=7.2, 1H), 6.73 (td, J=7.6, J=1.0, 1H), 6.25 (dd, J=7.6, J=1.0, 1H); ^{13}C NMR (75 MHz, DMSO-d_6) δ 164.1, 156.8, 152.2, 148.2, 135.8, 131.8, 131.3, 126.1, 125.6, 122.7, 122.5, 122.2, 116.5, 115.1, 112.6
Isomer Z: ^1H NMR (300 MHz, DMSO-d_6) δ 10.88 (broad s, 1H), 7.62-7.58 (m, 2H), 7.47 (td, J=7.2, J=1.2, 1H), 7.44 (dd, J=7.2, J=1.2, 1H), 7.32 (s, 1H), 7.28 (dd, J=7.2, J=1.2, 1H), 7.07 (td, J=7.2, J=1.2, 1H), 6.88 (d, J=7.8, 1H); ^{13}C NMR (75 MHz, DMSO-d_6) δ 159.4, 155.0, 150.8, 147.0, 135.5, 130.3, 130.1, 124.0, 125.3, 124.0, 123.8, 123.3, 121.9, 121.4, 111.8

(E,Z)-2-(4-(5,6-dioxane-2-oxoindoline-3-ylideneamino)phenyl)acetic acid (**DSF-091**). Compound **DSF-091** was prepared by reacting compound **19** with 2-(4-aminophenyl)acetic acid under microwave irradiation. Yield 18%.

Ratio E/Z: >95:5; mp = 266-268°C; IR (KBr): ṽ (cm^{-1}) = 3434 (NH), 1719 (C=O), 1327 (C=N), 1165 (C-O); HRMS (ESI-TOF) for $C_{18}H_{15}N_2O_5$ $(M+H)^+$: calcd = 339.0975, found = 339.1000
Isomer E: ^{1}H NMR (300 MHz, DMSO-d_6) δ = 12.35 (broad s, 1H), 10.69 (broad s, 1H), 7.36-7.31 (m, 2H), 6.90-6.86 (m, 2H), 6.34 (s, 1H), 5.89 (s, 1H), 4.24-4.19 (m, 2H), 4.08-4.05 (m, 2H), 3.61 (s, 2H); ^{13}C NMR (75 MHz, DMSO-d_6) δ 173.7, 164.9, 156.2, 150.2, 148.2, 147.0, 137.8, 132.4, 132.2, 117.0, 114.8, 113.5, 102.7, 65.8, 64.0, 40.3
Isomer Z was not characterized due the very low percentage in the mixture (less than 5%)

(E,Z)-2-(4-(6-chloro-5-fluoro-2-oxoindolin-3-ylideneamino)phenyl)acetic acid (**DSF-092**). Compound **DSF-092** was prepared by reacting compound **20** with 2-(4-aminophenyl)acetic acid under microwave irradiation. Yield 19%.
Ratio E/Z: 78:22; mp = 247-250°C; IR (KBr): ṽ (cm^{-1}) = 3319 (NH), 1718 (C=O), 1471 (C-F); HRMS (ESI-TOF) for $C_{16}H_{11}ClFN_2O_3$ $(M+H)^+$: calcd = 333.0437, found = 333.0444
Isomer E: ^{1}H NMR (300 MHz, DMSO-d_6) δ 12.34 (broad s, 1H), 11.15 (broad s, 1H), 7.41-7.36 (m, 2H), 7.06 (d, J=6.1, 1H), 7.00-6.95 (m, 2H), 6.23 (d, J=8.9, 1H), 3.65 (s, 2H); ^{13}C NMR (75 MHz, DMSO-d_6) δ 173.2, 163.8, 154.1, 152.5, 148.6, 144.4, 133.0, 131.2, 125.7, 117.8, 115.3, 113.4, 113.3, 40.6
Isomer Z: ^{1}H NMR (300 MHz, DMSO-d_6) δ 12.34 (broad s, 1H), 11.02 (broad s, 1H), 7.65 (d, J=8.1, 1H), 7.24-7.20 (m, 2H), 7.05-7.02 (m, 2H), 7.01 (d, J=6.4, 1H), 3.65 (s, 2H); ^{13}C NMR (75 MHz, DMSO-d_6) δ 173.2, 163.8, 158.2, 154.0, 148.6, 145.4, 134.5, 129.7, 126.2, 120.2, 115.4, 112.5, 111.4, 40.6

(E,Z)-2-(4-(5-methyl-2-oxoindolin-3-ylideneamino)phenyl)acetic acid (**DSF-093**). Compound **DSF-093** was prepared by reacting compound **21** with 2-(4-aminophenyl)acetic acid under conventional heating. Yield 52%.
Ratio E/Z: 85:15; mp = 231-232°C; IR (KBr): ṽ (cm^{-1}) = 3283 (NH), 1709 (C=O), 1220 (C=O); HRMS (ESI-TOF) for $C_{17}H_{15}N_2O_3$ $(M+H)^+$: calcd = 295.1077, found = 295.1054
Isomer E: ^{1}H NMR (300 MHz, DMSO-d_6) δ 12.34 (broad s, 1H), 10.86 (broad s, 1H), 7.37-7.33 (m, 2H), 7.16 (dd, J=8.0, J=1.8, 1H), 6.93-6.88 (m, 2H), 6.78 (d, J=8.0, 1H), 6.14 (d, J=1.8, 1H), 3.64 (s, 2H), 1.94 (s, 3H); ^{13}C NMR (75 MHz, DMSO-d_6) δ 173.3, 164.0, 155.8, 149.7, 145.1, 135.2, 132.3, 131.7, 131.1, 126.3, 122.0, 117.7, 111.7, 40.6, 20.9
Isomer Z: ^{1}H NMR (300 MHz, DMSO-d_6) δ 12.34 (broad s, 1H), 10.76 (broad s, 1H), 7.41 (d, J=1.1, 1H), 7.25 (dd, J=7.9, J=1.1, 1H), 7.21-7.18 (m, 2H), 6.98-6.94 (m, 2H), 6.75 (d, J=7.9, 1H), 3.55 (s, 2H), 2.28 (s, 3H); ^{13}C NMR (75 MHz, DMSO-d_6) δ 173.3, 159.1, 153.3, 147.8, 143.7, 134.9, 131.8, 130.9, 129.7, 123.5, 121.4, 119.8, 110.9, 40.6, 19.0

(E,Z)-2-(4-(5,7-dimethyl-2-oxoindolin-3-ylideneamino)phenyl)acetic acid (**DSF-094**). Compound **DSF-094** was prepared by reacting compound **22** with 2-(4-aminophenyl)acetic acid under conventional heating. Yield 26%.
Ratio E/Z: 84:16; mp = 240-242°C; IR (KBr): ṽ (cm^{-1}) = 3180 (NH), 1725 (C=O), 1617 (C=C), 1319 (C-N); HRMS (ESI-TOF) for $C_{18}H_{17}N_2O_3$ $(M+H)^+$: calcd = 309.1234, found = 309.1212
Isomer E: ^{1}H NMR (300 MHz, DMSO-d_6) δ 10.89 (broad s, 1H), 7.36-7.32 (m, 2H), 7.00 (s, 1H), 6.90-6.86 (m, 2H), 5.99 (s, 1H), 3.62 (s, 2H), 2.14 (s, 3H), 1.91 (s, 3H); ^{13}C NMR (75 MHz, DMSO-d_6) δ 173.5, 164.6, 156.1, 149.6, 143.7, 140.6, 136.5, 131.0, 130.9, 130.8, 123.7, 118.0, 117.6, 40.6, 20.2, 16.3
Isomer Z: ^{1}H NMR (300 MHz, DMSO-d_6) δ 10.81 (broad s, 1H), 7.24 (s, 1H), 7.22-7.18 (m, 2H), 7.09 (s, 1H), 6.96-6.92 (m, 2H), 3.54 (s, 2H), 2.25 (s, 3H), 2.22 (s, 3H); ^{13}C NMR (75 MHz, DMSO-d_6) δ 173.7, 160.5, 159.7, 147.5, 142.3, 136.5, 136.3, 131.8, 130.1, 129.7, 120.9, 119.8, 114.3, 40.7, 20.8, 16.0

(E,Z)-2-(4-(5-nitro-2-oxoindolin-3-ylideneamino)phenyl)acetic acid (**DSF-095**). Compound **DSF-095** was prepared by reacting compound **23** with 2-(4-aminophenyl)acetic acid under microwave irradiation. Yield 38%.
Ratio E/Z: 75:25; mp = 235-238°C; IR (KBr): ṽ (cm^{-1}) = 3225 (NH), 1735 (C=O), 1617 (C=C), 1343 (N=O); HRMS (ESI-TOF) for $C_{16}H_{12}N_3O_5$ $(M+H)^+$: calcd = 326.0771, found = 326.0785

Isomer E: ^{1}H NMR (300 MHz, DMSO-d_6) δ 8.27 (dd, J=8.7, J=2.4, 1H), 7.44-7.40 (m, 2H), 7.36 (d, J=2.4, 1H), 7.09 (d, J=8.7, 1H), 7.05-7.01 (m, 2H), 3.66 (s, 2H); ^{13}C NMR (75 MHz, DMSO-d_6) δ 173.0, 164.3, 152.8, 151.2, 148.4, 142.0, 133.2, 131.1, 130.6, 128.7, 120.9, 118.1, 112.2, 40.6

Isomer Z: ^{1}H NMR (300 MHz, DMSO-d_6) δ 8.37 (dd, J=8.6, J=2.4, 1H), 8.28 (d, J=2.4, 1H), 7.27-7.23 (m, 2H), 7.14-7.10 (m, 2H), 7.07 (d, J=8.6, 1H), 3.58 (s, 2H); ^{13}C NMR (75 MHz, DMSO-d_6) δ 173.1, 159.2, 153.6, 151.5, 146.8, 143.0, 133.0, 130.3, 129.8, 128.9, 120.6, 117.5, 111.5, 40.6

(E,Z)-2-(4-(5-methoxy-2-oxoindolin-3-ylideneamino)phenyl)acetic acid (**DSF-096**). Compound **DSF-096** was prepared by reacting compound **24** with 2-(4-aminophenyl)acetic acid under conventional heating. Yield 52%.

Ratio E/Z: 86:14; mp = 223-225°C; IR (KBr): ṽ (cm^{-1}) = 3235 (NH), 1742 (C=O), 1484 (C=C), 1306 (C-N); HRMS (ESI-TOF) for $C_{17}H_{15}N_2O_4$ (M+H)$^+$: calcd = 311.1026, found = 311.1038

Isomer E: ^{1}H NMR (300 MHz, DMSO-d_6) δ 12.34 (broad s, 1H), 10.78 (broad s, 1H), 7.38-7.34 (m, 2H), 6.98-6.90 (m, 3H), 6.81 (d, J=8.5, 1H), 5.91 (d, J=2.6, 1H), 3.62 (s, 2H), 3.39 (s, 3H); ^{13}C NMR (75 MHz, DMSO-d_6) δ 173.3, 164.1, 154.4, 149.5, 140.9, 132.4, 131.0, 127.0, 120.5, 116.5, 112.6, 111.5, 55.6, 40.9

Isomer Z: ^{1}H NMR (300 MHz, DMSO-d_6) δ 12.34 (broad s, 1H), 10.67 (broad s, 1H), 7.22-7.18 (m, 2H), 7.08 (d, J=2.6, 1H), 7.03 (dd, J=8.5, J=2.6, 1H), 6.99-6.95 (m, 2H), 6.78 (d, J=8.5, 1H), 3.76 (s, 3H), 3.55 (s, 2H); ^{13}C NMR (75 MHz, DMSO-d_6) δ 173.3, 163.4, 154.4, 149.5, 139.7, 131.8, 130.5, 129.8, 123.8, 120.9, 120.8, 112.5, 111.9, 55.6, 40.9

(E,Z)-3-(3-(trifluoromethyl)benzylidene)-5-bromoindolin-2-one (**DSF-097**). Compound **DSF-097** was prepared by reacting compound **18** with 3-(trifluoromethyl)aniline under conventional heating. Yield 30%.

Ratio E/Z: 73:27; mp = 151-153°C; IR (KBr): ṽ (cm^{-1}) = 3433 (NH), 1732 (C=O), 1316 (C-F); HRMS (ESI-TOF) for $C_{15}H_9BrF_3N_2O$ (M+H)$^+$: calcd = 368.9845, found = 368.9839

Isomer E: ^{1}H NMR (300 MHz, DMSO-d_6) δ 10.05 (broad s, 1H), 7.76-7.74 (m, 1H), 7.63-7.59 (m, 1H), 7.58 (dd, J=8.2, J=2.1, 1H), 7.39-7.37 (m, 1H), 7.25-7.23 (m, 1H), 6.99 (d, J=8.2, 1H), 6.54 (d, J=2.1, 1H); ^{13}C NMR (75 MHz, DMSO-d_6) δ 162.9, 158.2, 151.6, 146.7, 137.4, 132.7, 131.4, 128.5, 125.6, 122.3, 121.5, 118.0, 115.2, 114.0, 113.2

Isomer Z: ^{1}H NMR (300 MHz, DMSO-d_6) δ 9.95 (broad s, 1H), 7.78 (d, J=2.1, 1H), 7.68 (dd, J=8.2, J=2.1, 1H), 7.54 (dt, J=7.9, J=0.7, 1H), 7.45 (dt, J=7.9, J=0.7, 1H), 7.36-7.34 (m, 1H), 7.31-7.27 (m, 1H), 7.03 (d, J=8.2, 1H); ^{13}C NMR (75 MHz, DMSO-d_6) δ 164.2, 157.7, 150.3, 145.4, 137.1, 130.3, 130.3, 126.0, 125.8, 123.9, 121.4, 123.8, 117.9, 114.0, 113.8

(E,Z)-3-(3-(Trifluoromehyl)phenylimino)-5,6-dioxanoindolin-2-one (**DSF-098**). Compound **DSF-098** was prepared by reacting compound **19** with 3-(trifluoromethyl)aniline under microwave irradiation. Yield 15%.

Ratio E/Z: 69:31; mp = 279-281°C; IR (KBr): ṽ (cm^{-1}) = 3431 (NH), 1733 (C=O), 1322 (C-F); HRMS (ESI-TOF) for $C_{17}H_{12}F_3N_2O_3$ (M+H)$^+$: calcd = 349.0795, found = 349.0801

Isomer E: ^{1}H NMR (300 MHz, DMSO-d_6) δ 10.24 (broad s, 1H), 7.74-7.72 (m, 1H), 7.63 (d, J=7.9, 1H), 7.41 (s, 1H), 7.37 (d, J=7.9, 1H), 6.23 (s, 1H), 5.89 (s, 1H), 4.22-4.20 (m, 2H), 4.08-4.06 (m, 2H); ^{13}C NMR (75 MHz, DMSO-d_6) δ 158.0, 150.2, 147.2, 146.6, 146.2, 137.8, 131.4, 130.9, 129.9, 124.5, 114.8, 114.7, 113.5, 112.5, 102.4, 65.8, 64.0

Isomer Z: ^{1}H NMR (300 MHz, DMSO-d_6) δ 10.15 (broad s, 1H), 7.54-7.52 (m, 1H), 7.44 (d, J=7.9, 1H), 7.33 (s, 1H), 7.28 (d, J=7.9, 1H), 6.25 (s, 1H), 5.72 (s, 1H), 4.72-4.70 (m, 2H), 4.25-4.23 (m, 2H); ^{13}C NMR (75 MHz, DMSO-d_6) δ 158.0, 149.3, 147.0, 146.2, 145.3, 136.3, 129.7, 124.6, 122.6, 122.1, 121.9, 121.5, 115.3, 112.9, 102.6, 66.8, 63.2

(E,Z)-3-(3-(trifluoromethyl)benzylidene)-6-chloro-5-fluoroindolin-2-one (**DSF-099**). Compound **DSF-099** was prepared by reacting compound **20** with 3-(trifluoromethyl)aniline under conventional heating. Yield 30%.

RatioE/Z: 73:27; mp = 152-154°C; IR (KBr): ṽ (cm^{-1}) = 3178 (NH), 1741 (C=O), 1618 (C=N), 1473 (C=C), 1330 (C-F); HRMS (ESI-TOF) for $C_{15}H_8ClF_4N_2O$ $(M+H)^+$: calcd = 343.0256, found = 343.0251
Isomer E: ^1H NMR (300 MHz, DMSO-d_6) δ 11.15 (broad s, 1H), 7.78-7.76 (t, J=7.7, 1H), 7.70 (d, J=7.7, 1H), 7.46 (s, 1H), 7.39 (d, J=7.7, 1H), 7.16 (d, J=6.1, 1H), 6.18 (d, J=8.9, 1H);^{13}C NMR (75 MHz, DMSO-d_6) δ 164.2, 155.2, 153.5, 148.6, 145.4, 131.6, 130.9, 126.7, 124.6, 122.4, 122.2, 116.3, 114.8, 113.3, 112.5
Isomer Z: ^1H NMR (300 MHz, DMSO-d_6) δ 11.03 (broad s, 1H), 7.65 (d, J=8.1, 1H), 7.59-7.57 (t, J=8.2, 1H), 7.49 (d, J=8.2, 1H), 7.44 (s, 1H), 7.36 (d, J=8.2, 1H), 7.01 (d, J=6.4, 1H); ^{13}C NMR (75 MHz, DMSO-d_6) δ 163.0, 158.2, 154.0, 148.6, 145.4, 130.8, 129.7, 126.2, 124.4, 123.0, 121.7, 116.3, 115.4, 112.5, 111.4

(E,Z)-3-(3-(trifluoromethyl)phenylimino)-5-methylindolin-2-one (**DSF-100**). Compound **DSF-100** was prepared by reacting compound **21** with 3-(trifluoromethyl)aniline under conventional. Yield 33%.
Ratio E/Z: 64:36; mp = 230-232°C; IR (KBr): ṽ (cm^{-1}) = 3268 (NH), 1739 (C=O), 1617 (C=N), 1333 (C-F); HRMS (ESI-TOF) for $C_{16}H_{12}F_3N_2O$ $(M+H)^+$: calcd = 305.0896, found = 305.0900
Isomer E: ^1H NMR (300 MHz, DMSO-d_6) δ 10.91 (broad s, 1H), 7.71 (t, J=7.8, 1H), 7.63 (d, J=7.8, 1H), 7.39 (s, 1H), 7.26 (d, J=7.8, 1H), 7.19 (dd, J=8.0, J=1.0, 1H), 6.81 (d, J=8.0, 1H), 6.05 (d, J=1.0, 1H), 1.94 (s, 3H); ^{13}C NMR (75 MHz, DMSO-d_6) δ 163.8, 156.6, 151.6, 145.6, 135.7, 131.9, 131.4, 131.2, 126.0, 124.5, 123.4, 121.9, 116.1, 114.9, 112.0, 20.2
Isomer Z: ^1H NMR (300 MHz, DMSO-d_6) δ 10.79 (broad s, 1H), 7.53 (t, J=7.8, 1H), 7.42-7.40 (m, 1H), 7.34-7.30 (m, 2H), 7.27-7.25 (m, 2H), 6.77 (d, J=8.0, 1H), 2.29 (s, 3H); ^{13}C NMR (75 MHz, DMSO-d_6) δ 159.2, 154.8, 150.5, 144.3, 135.5, 130.9, 130.8, 129.9, 124.7, 123.4, 122.7, 122.4, 121.6, 114.8, 111.2, 20.2

(E,Z)-3-(3-(trifluoromethyl)phenylimino)-5,7-dimethylindolin-2-one (**DSF-101**). Compound **DSF-101** was prepared by reacting compound **22** with 3-(trifluoromethyl)aniline under conventional heating. Yield 29%.
Ratio E/Z: 64:36; mp = 240-242°C; IR (KBr): ṽ (cm^{-1}) = 3432 (NH), 1725 (C=O), 1334 (C-F); HRMS (ESI-TOF) for $C_{17}H_{14}F_3N_2O$ $(M+H)^+$: calcd = 319.1053, found = 319.1061
Isomer E: ^1H NMR (300 MHz, DMSO-d_6) δ 10.94 (broad s, 1H), 7.71 (t, J=7.9, 1H), 7.62 (d, J=7.9, 1H), 7.36 (s, 1H), 7.31 (d, J=7.9, 1H), 7.04 (s, 1H), 5.89 (s, 1H), 2.15 (s, 3H), 1.91 (s, 3H); ^{13}C NMR (75 MHz, DMSO-d_6) δ 164.3, 156.9, 151.6, 144.2, 136.9, 131.8, 131.4, 129.2, 123.5, 122.7, 121.3, 115.8, 114.8, 20.7, 16.3
Isomer Z: ^1H NMR (300 MHz, DMSO-d_6) δ 10.82 (broad s, 1H), 7.53 (t, J=7.9, 1H), 7.43 (d, J=7.9, 1H), 7.31 (d, J=7.9, 1H), 7.26 (s, 1H), 7.24 (s, 1H), 7.13 (s, 1H), 2.26 (s, 3H), 2.16 (s, 3H); ^{13}C NMR (75 MHz, DMSO-d_6) δ 159.7, 155.2, 150.6, 142.9, 136.8, 130.9, 130.8, 130.0, 129.9, 123.8, 123.4, 121.7, 121.3, 120.5, 115.9, 20.7, 16.1

(E,Z)-3-(3-(trifluoromethyl)benzylidene)-5-nitroindolin-2-one (**DSF-102**). Compound **DSF-102** was prepared by reacting compound **23** with 3-(trifluoromethyl)aniline under microwave irradiation. Yield 27.8%.
Ratio E/Z: 65:35; mp = 250-252°C; IR (KBr): ṽ (cm^{-1}) = 3297 (NH), 1751 (C=O), 1618 (C=N), 1328 (C-F), 1164 (C-O), 1125 (N=O) cm-1.; HRMS (ESI-TOF) for $C_{15}H_9F_3N_3O_3$ $(M+H)^+$: calcd = 336.0591, found = 336.0579
Isomer E: ^1H NMR (300 MHz, DMSO-d_6) δ 8.30 (dd, J=8.8, J=2.3, 1H), 7.78 (t, J=7.8, 1H), 7.72 (d, J=7.8, 1H), 7.48 (s, 1H), 7.39 (d, J=7.8, 1H), 7.12-7.07 (m, 2H); ^{13}C NMR (75 MHz, DMSO-d_6) δ 165.4, 155.8, 150.9, 141.4, 139.7, 131.6, 131.0, 130.9, 124.6, 122.4, 122.0, 120.7, 114.8, 112.7
Isomer Z: ^1H NMR (300 MHz, DMSO-d_6) δ 8.40 (dd, J=8.7, J=2.5, 1H), 8.27 (d, J=2.5, 1H), 7.58 (t, J=7.9, 1H), 7.51 (d, J=7.9, 1H), 7.48-7.46 (m, 1H), 7.36 (d, J=7.9, 1H), 7.10-7.06 (m, 1H); ^{13}C NMR (75 MHz, DMSO-d_6) δ 160.6, 155.5, 149.7, 142.5, 137.5, 130.9, 130.8, 129.7, 124.6, 124.4, 123.0, 121.7, 118.3, 116.3, 111.9

(E,Z)-3-(3-(trifluoromethyl)phenylimino)-5-methoxyindolin-2-one (**DSF-103**). Compound **DSF-103** was prepared by reacting compound **24** with 3-(trifluoromethyl)aniline under conventional. Yield 58%.
Ratio E/Z: 66:34; mp = 236-239°C; IR (KBr): ṽ (cm^{-1}) = 3448 (NH), 1749 (C=O), 1629 (C=N), 1476 (C=C), 1339 (C-F), 1302 (C-N); HRMS (ESI-TOF) for $C_{16}H_{12}F_3N_2O_2$ $(M+H)^+$: calcd = 321.0845, found = 321.0829
Isomer E: ^{1}H NMR (300 MHz, DMSO-d_6) δ 10.82 (broad s, 1H), 7.73 (t, J=7.8, 1H), 7.63 (d, J=7.8, 1H), 7.41 (s, 1H), 7.33 (d, J=7.8, 1H), 6.99 (dd, J=8.5, J=2.6, 1H), 6.84 (d, J=8.5, 1H), 5.72 (d, J=2.6, 1H), 3.40 (s, 3H); ^{13}C NMR (75 MHz, DMSO-d_6) δ 163.8, 154.4, 151.5, 141.3, 131.4, 130.8, 123.0, 124.5, 122.1, 120.6, 114.7, 112.8, 112.4, 108.3, 55.5
Isomer Z: ^{1}H NMR (300 MHz, DMSO-d_6) δ 10.69 (broad s, 1H), 7.53 (t, J=7.9, 1H), 7.44 (d, J=7.9, 1H), 7.33 (s, 1H), 7.28 (d, J=7.9, 1H), 7.18 (d, J=2.6, 1H), 7.06 (dd, J=8.5, J=2.6, 1H), 6.81 (d, J=8.5, 1H), 3.77 (s, 3H); ^{13}C NMR (75 MHz, DMSO-d_6) δ 159.2, 156.9, 150.3, 140.3, 129.9, 129.7, 124.6, 122.6, 122.1, 122.1, 121.9, 121.5, 115.9, 111.4, 56.2

2. Surface Plasmonic Resonance (SPR) experiments

2.1. EGFR-EGF interaction: SPR measurements were carried out at 25 °C on a dual flow-cell Biacore-X100 instrument (GE-Healthcare, Chicago IL). EGFR (SinoBiological, Beijing China) was immobilized (3360 RU) on a carboxymethylated-dextran chip (CM5), using the amide coupling chemistry at pH 4.0. Increasing concentrations of recombinant EGF (DiscoverX inc) were injected over the EGFR-coated sensor chip at a flow rate of 10 μL/min, with a contact time of 180 sec, in 10 mM Hepes pH 7.4, 150 mM NaCl, 3 mM EDTA, 0.005% polyoxyethylene sorbitan

Each binding curve was subtracted for the corresponding baseline obtained on the reference flow cell and accounting for nonspecific binding, which was found <2% of RU_{max}. The dissociation constant (K_d) relative to the binding of the ligand (L), *i.e.* EGF, to the immobilized receptor (R), *i.e.* EGFR, was obtained as a fitting parameter by plotting the value of the response units at the steady state (RU_{eq}), after reaching equilibrium at each ligand concentration (Figure S2), and fitting the data points with an equation describing the one-site binding model:

$$RUeq = RUmax \cdot \frac{[L]_F}{[L]_F + K_d}$$

where RU_{max} is the RU value at saturating L concentrations and $[L]_F$ is the concentration of the free ligand in equilibrium with the receptor-ligand (RL) complex present on the sensor chip surface. When $[R] << K_d$, then $[L]_F$ can be approximated to $[L]_T$, i.e. the total ligand concentration in the mobile phase. Data analysis was performed using the BIAevaluation software and OriginPro 2018b.

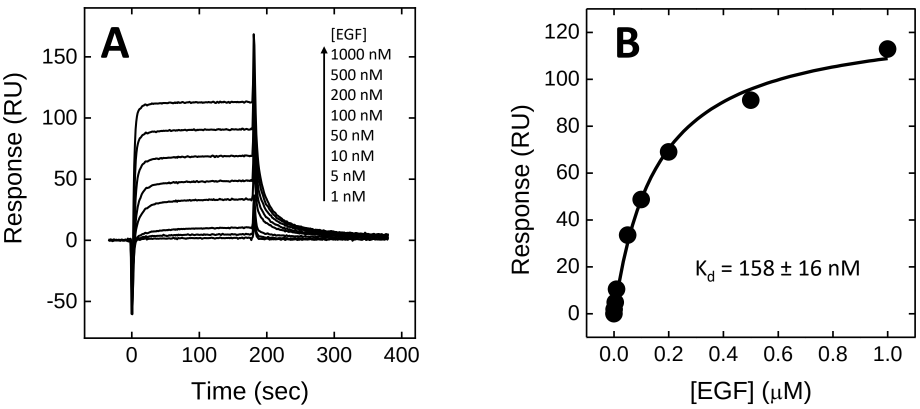

Figure S1. Binding of EGF to EGFR monitored by surface plasmon resonance. (**A**) Sensograms relative to EGF binding. (**B**) Plot of RU_{max} versus EGF concentration. The fitting of data points with one-binding equation yields a K_d value of 158 ± 16 nM.

2.2. DSF-102 competition experiments: Competition assays with **DSF-102** in solution were carried out by incubating a constant concentration of EGF (1 μM) with increasing amounts of **DSF-102** up to 100 μM for 30 min, followed by injection over the EGFR sensor chip. All measurements were carried out at a flow rate of 10 μl/min, at 25°C using 10 mM Hepes pH 7.4, 150 mM NaCl, 3 mM EDTA, 0.005% polyoxyethylene sorbitan as running buffer. The SPR signal was taken at the end of the association phase and plotted against **DSF-102** concentration in order to visualize the effect of competition.

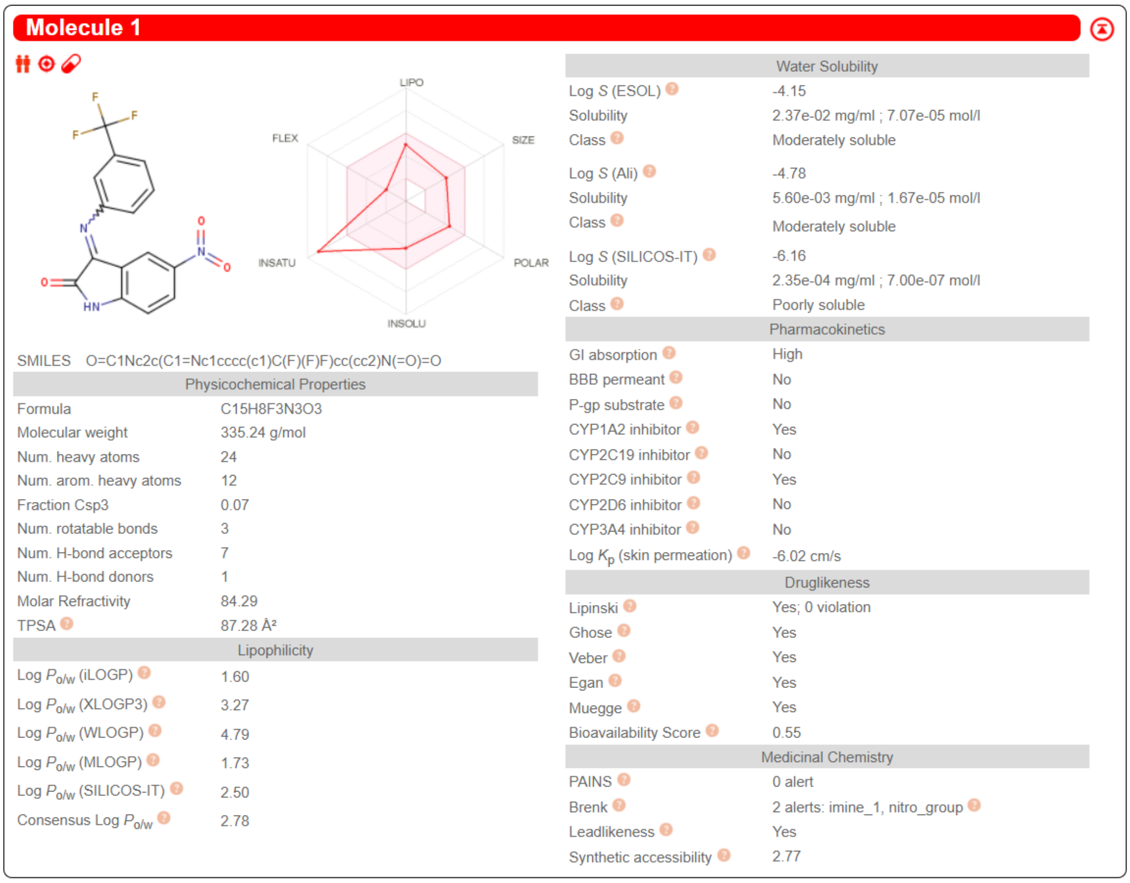

Molecule 1

SMILES O=C1Nc2c(C1=Nc1cccc(c1)C(F)(F)F)cc(cc2)N(=O)=O

Physicochemical Properties	
Formula	C15H8F3N3O3
Molecular weight	335.24 g/mol
Num. heavy atoms	24
Num. arom. heavy atoms	12
Fraction Csp3	0.07
Num. rotatable bonds	3
Num. H-bond acceptors	7
Num. H-bond donors	1
Molar Refractivity	84.29
TPSA	87.28 Å²

Lipophilicity	
Log $P_{o/w}$ (iLOGP)	1.60
Log $P_{o/w}$ (XLOGP3)	3.27
Log $P_{o/w}$ (WLOGP)	4.79
Log $P_{o/w}$ (MLOGP)	1.73
Log $P_{o/w}$ (SILICOS-IT)	2.50
Consensus Log $P_{o/w}$	2.78

Water Solubility	
Log *S* (ESOL)	-4.15
Solubility	2.37e-02 mg/ml ; 7.07e-05 mol/l
Class	Moderately soluble
Log *S* (Ali)	-4.78
Solubility	5.60e-03 mg/ml ; 1.67e-05 mol/l
Class	Moderately soluble
Log *S* (SILICOS-IT)	-6.16
Solubility	2.35e-04 mg/ml ; 7.00e-07 mol/l
Class	Poorly soluble

Pharmacokinetics	
GI absorption	High
BBB permeant	No
P-gp substrate	No
CYP1A2 inhibitor	Yes
CYP2C19 inhibitor	No
CYP2C9 inhibitor	Yes
CYP2D6 inhibitor	No
CYP3A4 inhibitor	No
Log K_p (skin permeation)	-6.02 cm/s

Druglikeness	
Lipinski	Yes; 0 violation
Ghose	Yes
Veber	Yes
Egan	Yes
Muegge	Yes
Bioavailability Score	0.55

Medicinal Chemistry	
PAINS	0 alert
Brenk	2 alerts: imine_1, nitro_group
Leadlikeness	Yes
Synthetic accessibility	2.77

Figure S2. Drug likeness properties of DSF-102. Drug likeness properties have been computed using the SwissADME online tool [5].

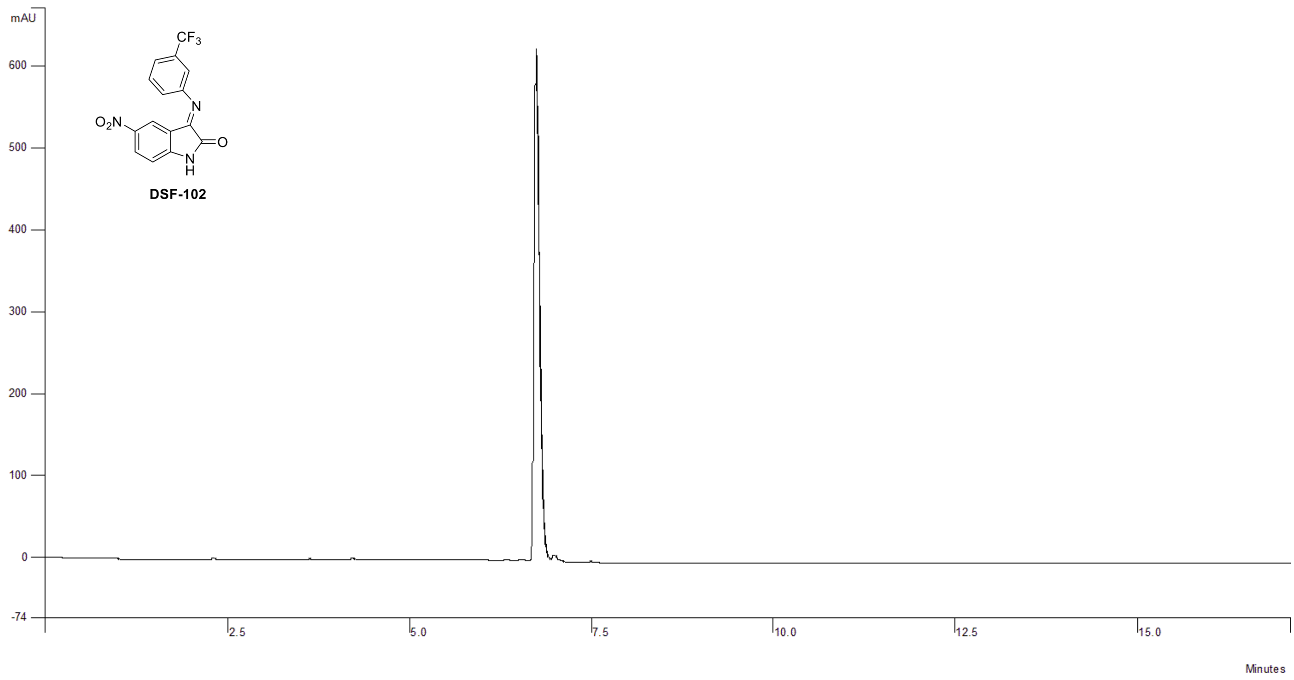

Figure S3. HPLC analysis of DSF-102

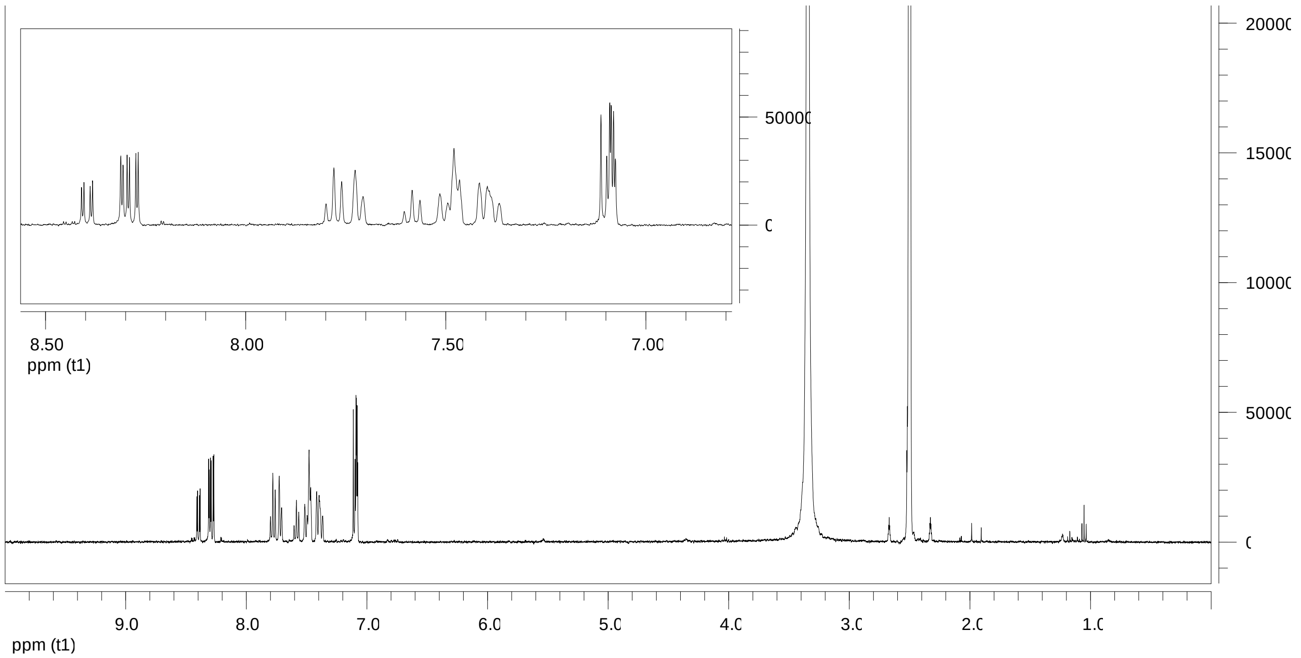

Figure S4. 1H-NMR of DSF-102

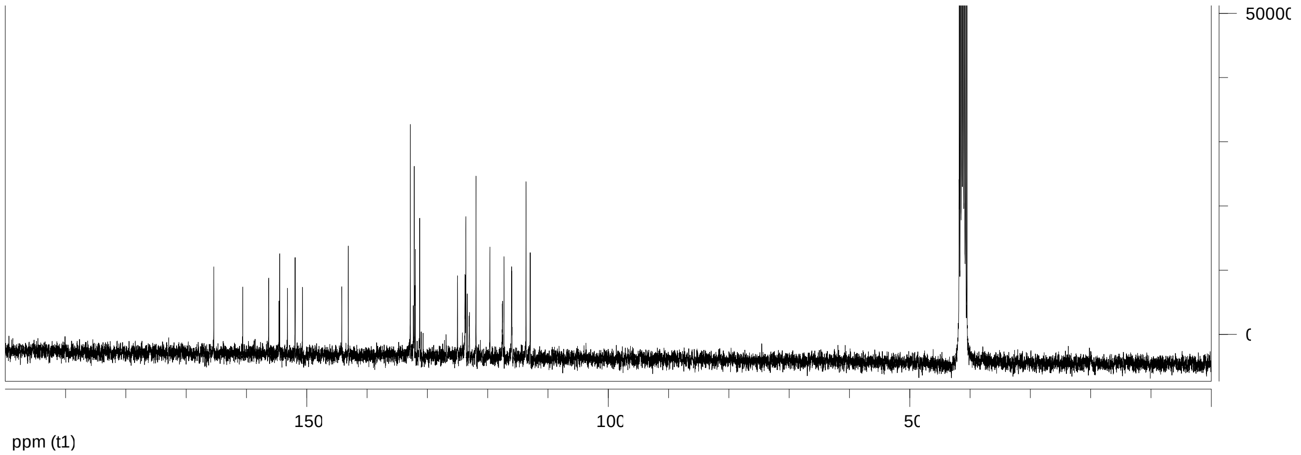

Figure S5. 13C-NMR of DSF-102

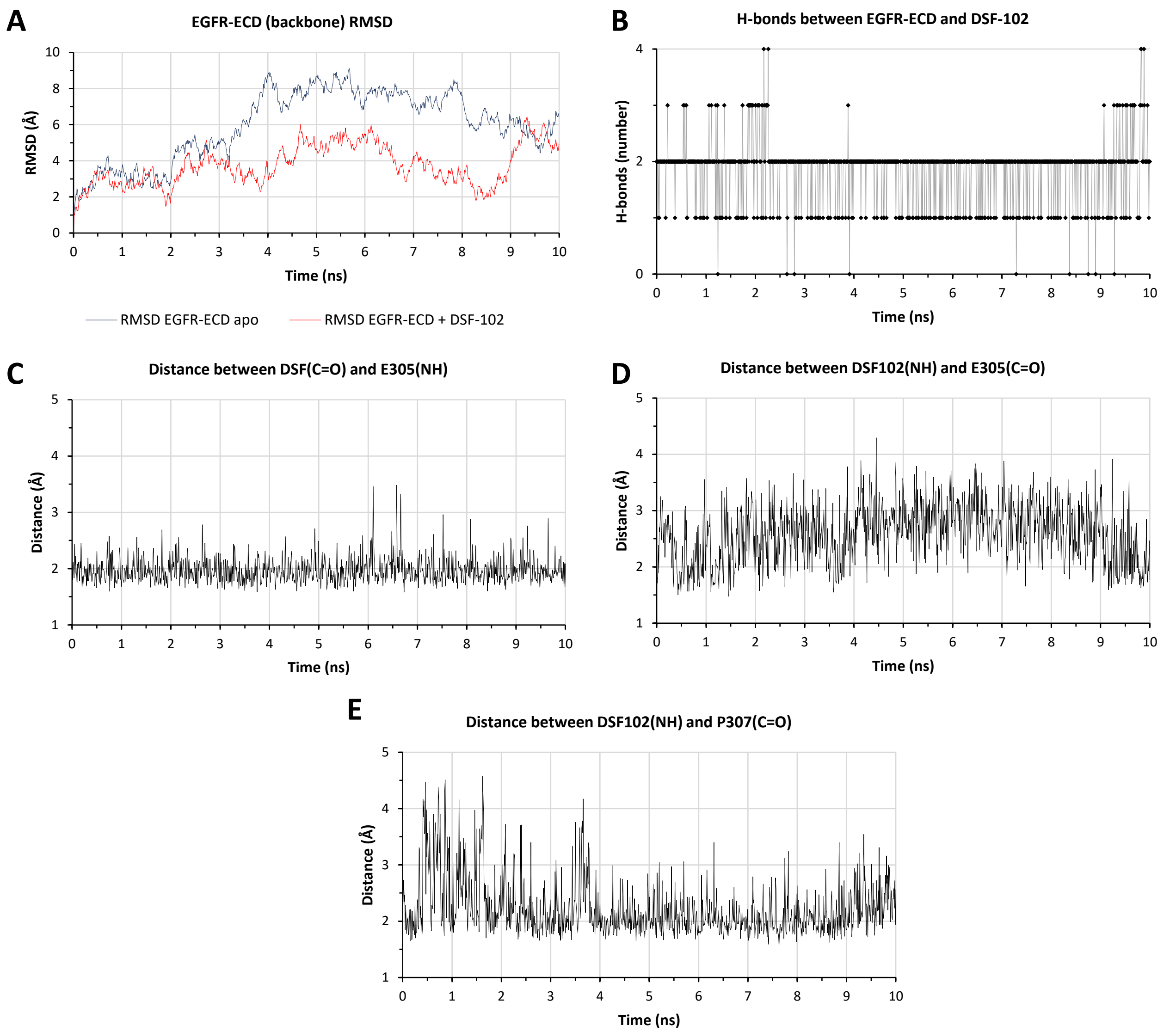

Figure S6. Results from MD studies. (**A**) RMSD (Å) of apo (blue line) and **DSF-102** bound (red line) EGFR-ECD during 10 ns of simulations. (**B**) Number of H-bonds formed between **DSF-102** and EGFR-ECD calculated through the hbond utility of Gromacs software. (**C**) Distance (Å) between **DSF-102** C=O and ^{305}Glu amide-NH. The distance was computed between the oxygen and the hydrogen atoms. (**D**) Distance (Å) between **DSF-102** NH and ^{305}Glu amide-C=O. The distance was computed between the hydrogen and the oxygen atoms. (**E**) Distance (Å) between **DSF-102** NH and ^{307}Pro amide-C=O. The distance was computed between the hydrogen and the oxygen atoms.

Table S1. List of screened compounds, SMILES code and % of inhibition at 50 and 10 μM (mean ± SD of three independent experiments)

Compound	SMILES	% inhibition @ 50 uM	% inhibition @ 10 uM
**DSF001**	NC1=CC2=CC3=C(C=C(N)C=C3)C=C2C=C1	0 ± 2	0 ± 2
**DSF002**	CC(C1=CC=CC2=C1C=C(C=CC=C3C(C)=O)C3=C2)=O	0 ± 2	0 ± 3
**DSF003**	ClCC1=C2C(C=CC=C2)=C(CCl)C3=C1C=CC=C3	50 ± 1	35 ± 5
**DSF004**	N#CCC1=C2C(C=CC=C2)=C(CC#N)C3=C1C=CC=C3	1 ± 1	0 ± 2
**DSF005**	O=C(C1=CC=CC2=C1C=C(C=CC=C3C(O)=O)C3=C2)O	1 ± 4	0 ± 5
**DSF006**	C1(C=CC=C2)=C2C(CN3CCOCC3)=C4C=CC=CC4=C1CN5CCOCC5	27 ± 4	19 ± 1
**DSF007**	C1(C=CC=C2)=C2C(CN3CCCCC3)=C4C=CC=CC4=C1CN5CCCCC5	16 ± 4	16 ± 4
**DSF008**	O=C(C1=C2C=CC=C1)C3=C(C(NC(C)=O)=CC=C3O)C2=O	14 ± 2	0 ± 5
**DSF009**	O=C(C1=C2C(NCCOCCN)=CC=C1NCCOCCN)C3=C(C=CC(C(NC4=<br>CC=C(NS(=O)(C)=O)C=C4)=O)=C3)C2=O	48 ± 1	8 ± 1
**DSF010**	O=C(C1=C2C(NCCN3CCNCC3)=CC=C1NCCN4CCNCC4)C5=C(C=C<br>C(C(NC6=CC=C(NS(=O)(C)=O)C=C6)=O)=C5)C2=O	34 ± 2	28 ± 3
**DSF011**	O=C(C1=C2C=CC=C1O)C3=C(C=C(COC(COCC(O)=O)=O)C=C3O)C2=<br>O	39 ± 1	9 ± 5
**DSF012**	O=C(C1=C2C=CC=C1)C3=C(C(NCCCCCOC(C(C)NC(C(C(C)C)NC(O<br>CC4=CC=CC=C4)=O)=O)=O)=CC=C3NCCCCCOC(C(C)NC(C(C(C)C)<br>NC(OCC5=CC=CC=C5)=O)=O)=O)C2=O	20 ± 5	0 ± 1
**DSF013**	O=C(C1=C2C=CC=C1)C3=C(C(NCCCCCO)=CC=C3NCCCCCOC(C(C)<br>NC(C(C(C)C)NC(OCC4=CC=CC=C4)=O)=O)=O)C2=O	0 ± 2	0 ± 5
**DSF014**	O=C(C1=C2C=CC=C1OCC=C)C3=C(C(OCC=C)=CC=C3)C2=O	0 ± 1	0 ± 3
**DSF015**	O=C(C1=C2C=CC=C1)C3=C(C(F)=CC=C3NCCN(C)C)C2=O	48 ± 1	39 ± 4
**DSF016**	O=C(C1=C2C=CC=C1)C3=C(C(F)=CC=C3NCCNCCO)C2=O	25 ± 3	33 ± 5
**DSF017**	O=C(C1=C2C=CC=C1)C3=C(C(NC(CN4CCCCC4)=O)=CC=C3NC(CN<br>5CCCCC5)=O)C2=O	14 ± 1	9 ± 2
**DSF018**	O=C(C1=C2C=CC=C1)C3=C(C(NC(CN4CCOCC4)=O)=CC=C3NC(CN<br>5CCOCC5)=O)C2=O	38 ± 5	16 ± 3
**DSF019**	O=C(C1=C2C(O)=CC=C1O)C3=C(C(NC(CN4CCCCC4)=O)=CC=C3NC<br>(CN5CCCCC5)=O)C2=O	33 ± 1	35 ± 5
**DSF020**	O=C(C1=C2C(O)=CC=C1O)C3=C(C(NC(CN4CCOCC4)=O)=CC=C3NC<br>(CN5CCOCC5)=O)C2=O	29 ± 1	33 ± 5
**DSF021**	O=C(C1=C2C=CC=C1)C3=C(C(NC(CCN4CCOCC4)=O)=CC=C3NC(C<br>CN5CCOCC5)=O)C2=O	30 ± 5	16 ± 4
**DSF022**	NC1=CC=CC(C2=NC=NC(C3=CC=CC=C3)=C2)=C1	4 ± 4	0 ± 2
**DSF023**	NC1=CC=CC(C2=NC=NC(C3=CC=C(C(O)=O)C=C3)=C2)=C1	8 ± 2	22 ± 1
**DSF024**	OC1=NC(C2=CC=C(C(O)=O)C=C2)=CC(C3=CC(C(F)(F)F)=CC=C3)=N1	5 ± 3	16 ± 1
**DSF025**	FC(C1=CC=CC(C2=NC=NC(C3=CC=C(C(O)=O)C=C3)=C2)=C1)(F)F	34 ± 1	6 ± 5
**DSF026**	FC(C1=CC=CC(C2=NC=NC(C3=CC=CC=C3)=C2)=C1)(F)F	0 ± 1	0 ± 5
**DSF027**	C1(C2=CC=CC=C2)=CC(C3=CC(C4=CC=CC=C4)=CC=C3)=NC=N1	0 ± 1	0 ± 2
**DSF028**	BrC1=CC=CC(C2=NC=NC(C3=CC=CC=C3)=C2)=C1	0 ± 1	0 ± 4
**DSF029**	OC1=CC(C2=CC=CC=C2)=CC=C1C3=CC=C(C(O)=O)C=C3	0 ± 3	1 ± 4
**DSF030**	OC1=CC(C2=CC=CC=C2)=CC=C1C3=CC=C(C(NCC4=CC=CC=C4)=O)<br>C=C3	0 ± 1	0 ± 5
**DSF031**	O=C1OC2=C(C=CC(NC3=CC=CC(OC)=C3)=N2)C=C1C4=CC=CS4	19 ± 1	0 ± 1
**DSF032**	O=C1OC2=C(C=CC(NC3=CC=CC(SC)=C3)=N2)C=C1C4=CC=CS4	3 ± 5	0 ± 3

**DSF033**	O=C1OC2=C(C(N(C)C)=NC(NC3=CC=CC(OC)=C3)=N2)C=C1C4=C(Cl)C=CC=C4Cl	12 ± 3	0 ± 2
**DSF034**	O=C1OC2=C(C(O)=NC(NC3=CC=CC(OC)=C3)=N2)C=C1C4=C(Cl)C=CC=C4Cl	0 ± 1	14 ± 2
**DSF035**	O=C1OC2=C(C(O)=NC(NC3=CC=CC(OC)=C3)=N2)C=C1C4=CC(OC)=CC(OC)=C4	0 ± 2	0 ± 5
**DSF036**	O=C1OC2=C(C(O)=NC(NC3=CC=CC(OC)=C3)=N2)C=C1C4=CC=CC=C4	0 ± 5	0 ± 3
**DSF037**	O=C1OC2=C(C(NC3=CC=CC(C4=CC=CC=C4)=C3)=NC=N2)C=C1C5=C(Cl)C=CC=C5Cl	40 ± 5	12 ± 4
**DSF038**	O=C1OC2=C(C(NC3=CC=CC(C)=C3)=NC=N2)C=C1C4=C(Cl)C=CC=C4Cl	24 ± 5	0 ± 1
**DSF039**	O=C1OC2=C(C(N3CCN(C)CC3)=NC=N2)C=C1C4=C(Cl)C=CC=C4Cl	10 ± 2	0 ± 2
**DSF040**	O=C1OC2=NC(O)=NC=C2C=C1C(OCC)=O	0 ± 5	0 ± 1
**DSF041**	O=C1OC2=NC(NC3=CC=CC(C4=CC=CC=C4)=C3)=NC(O)=C2C=C1C5=CC=CC(Br)=C5	0 ± 4	0 ± 1
**DSF042**	OC1=CC=C2C(OC(C(CC(OC)=O)=C2C)=O)=C1	0 ± 2	0 ± 4
**DSF043**	O=C1OC2=CC(OC)=CC=C2C(C)=C1CC(NC3=CC=C(C(O)=O)C=C3)=O	0 ± 2	0 ± 2
**DSF044**	O=C1OC2=C(C)C(OC)=CC=C2C(C)=C1CCC(NC3=CC=C(C(O)=O)C=C3)=O	0 ± 1	0 ± 2
**DSF045**	O=C1OC2=CC(OC)=CC=C2C(C)=C1CCC(NC3=CC=C(C(O)=O)C=C3)=O	0 ± 5	0 ± 3
**DSF046**	O=C1OC2=CC(OCCOC)=CC=C2C(C)=C1CC(NC3=CC=C(C(O)=O)C=C3)=O	0 ± 1	0 ± 2
**DSF047**	O=C1OC2=C(C)C(OC)=CC=C2C(C)=C1CC(NC3=CC=CC(C(O)=O)=C3)=O	0 ± 1	0 ± 4
**DSF048**	O=C1OC2=CC(OCCOC)=CC=C2C(C)=C1CCC(NC3=CC=C(C(O)=O)C=C3)=O	3 ± 4	9 ± 3
**DSF049**	O=C1OC2=NC3=C(C(CCCC4)=C4O3)C=C2C(C)=C1CCC(NC5=CC(C(O)=O)=CC=C5)=O	36 ± 3	35 ± 2
**DSF050**	O=C1OC2=C(C)C3=C(C(CCCC4)=C4O3)C=C2C(C)=C1CC(NC5=CC=CC(O)=C5)=O	32 ± 2	24 ± 4
**DSF051**	O=C1OC2=C(C)C3=C(C(CCCC4)=C4O3)C=C2C(C)=C1CC(NC5=CC=C(NS(=O)(C)=O)C=C5)=O	50 ± 1	40 ± 1
**DSF052**	O=C1OC2=C(C)C3=C(C(CCCC4)=C4O3)C=C2C(C)=C1CC(NC5=CC=C(NC(C)=O)C=C5)=O	49 ± 2	41 ± 4
**DSF053**	O=C1OC2=C(C)C3=C(C(CCCC4)=C4O3)C=C2C(C)=C1CC(NC5=CC=C(C(N)=O)C=C5)=O	43 ± 1	39 ± 5
**DSF054**	O=C1OC2=C(O)C3=C(C(CCCC4)=C4O3)C=C2C(C)=C1C	46 ± 4	8 ± 4
**DSF055**	CC1=C(C)OC2=C1C=C3C(OC(C(CC(NCC4=CC=CC=C4Cl)=O)=C3C)=O)=C2C	46 ± 5	46 ± 1
**DSF056**	O=C(NC1=CC=C(C)C=C1)NC(C=C2)=CC=C2NC3=NC=NC(C4=CC=CC=C4)=C3	22 ± 5	0 ± 1
**DSF057**	O=C(NC1=CC=C(C(O)=O)C=C1)NC(C=C2)=CC=C2NC3=NC=NC(C4=CC=CC=C4)=C3	44 ± 5	17 ± 2
**DSF058**	OC1=NC(NC2=CC(OC)=CC=C2)=NC=C1C(O)=O	5 ± 5	12 ± 2
**DSF059**	CC1=CC=C([N+]([O-])=O)C=C1NC2=NC=CC(C3=CN=CC=C3)=N2	0 ± 5	0 ± 5
**DSF060**	ClC1=NC(C)=NC(NC2=NC=C(C(O)=O)S2)=C1	0 ± 2	0 ± 1
**DSF061**	ClC1=CN=C(OC2=CC=CC(NC(NC(C3=CC=CC=C3)=O)=O)=C2)N=C1	1 ± 1	20 ± 4
**DSF062**	ClC1=CN=C(OC2=CC=CC(NC(NC(C3=CC=CC=C3)=O)=S)=C2)N=C1	21 ± 2	9 ± 3

**DSF063**	ClC1=CN=C(OC2=CC=CC(NC(NC(C3=CC=CC=C3[N+]([O-])=O)=O)=O)=C2)N=C1	5 ± 5	0 ± 4
**DSF064**	O=C(C1=CC=CC=C1)NC(NC2=CC(OC3=NC=C(CC)C=N3)=CC=C2)=O	0 ± 2	0 ± 5
**DSF065**	O=C(C1=CC=CC=C1)NC(NC2=CC(OC3=NC=C(CC)C=N3)=CC=C2)=S	22 ± 5	0 ± 5
**DSF066**	O=C(C1=CC=CC=C1[N+]([O-])=O)NC(NC2=CC(OC3=NC=C(CC)C=N3)=CC=C2)=O	0 ± 4	0 ± 4
**DSF067**	O=C1NC2=CC=CC=C2/C1=N\C3=CC=C(CC(O)=O)C=C3	42 ± 1	42 ± 5
**DSF068**	O=C1NC2=CC=C(Br)C=C2/C1=N\C3=CC=C(CC(O)=O)C=C3	56 ± 4	37 ± 5
**DSF069**	O=C1NC2=CC=CC=C2/C1=N\C3=CC(C(F)(F)F)=CC=C3	56 ± 2	27 ± 5
**DSF070**	O=C1NC2=CC=C(Br)C=C2/C1=N\C3=C(OC)C=CC(OC)=C3	41 ± 1	20 ± 5
**DSF071**	O=C1OC(C2=CC(C(O)=O)=CC=C2)=N/C1=C/C3=CC=C(NC(C)=O)C=C3	0 ± 5	0 ± 3
**DSF072**	O=C1OC(C2=CC=CC=C2)=N/C1=C/C3=CC(OC)=C(OC(C)=O)C=C3	39 ± 2	16 ± 4
**DSF073**	O=C1OC(C2=CC=CC=C2)=N/C1=C/C3=CC=C(N(C)C)C=C3	36 ± 3	23 ± 1
**DSF074**	O=C1OC(C2=CC=CC=C2)=N/C1=C/C3=CN=C(C=CC=C4)C4=C3	48 ± 2	10 ± 3
**DSF075**	O=C1OC(C2=CC=CC=C2)=N/C1=C/C3=CC=CN3	34 ± 4	18 ± 5
**DSF076**	O=C1OC(C2=CC=CC=C2)=N/C1=C/C3=CC=C(OC)C=C3	18 ± 4	25 ± 2
**DSF077**	O=C1OC(C2=CC=CC=C2)=N/C1=C/C3=CN(C(C)=O)C4=C3C=CC=C4	39 ± 1	7 ± 1
**DSF078**	O=C1OC(C2=CC=CN=C2Cl)=N/C1=C/C3=CC=C(NC(C)=O)C=C3	37 ± 4	7 ± 1
**DSF079**	O=C1OC(C2=CC(OC)=CC=C2[N+]([O-])=O)=N/C1=C/C3=CC=C(NC(C)=O)C=C3	41 ± 2	3 ± 1
**DSF080**	O=C1OC(C2=CC=C([N+]([O-])=O)C=C2O)=N/C1=C/C3=CC=C(NC(C)=O)C=C3	28 ± 3	11 ± 5
**DSF081**	O=S(C1=CC=C(CC(O)=O)C=C1)(NC2=CC=CC=C2)=O	26 ± 1	21 ± 5
**DSF082**	CC(C=CC=C1C)=C1NS(C2=CC=C(CC(O)=O)C=C2)(=O)=O	19 ± 5	16 ± 1
**DSF083**	CC(C=CC=C1C)=C1NS(C2=CC=C(CC(O)=O)C=C2)(=O)=O	49 ± 3	36 ± 4
**DSF084**	ClC1=CC([N+]([O-])=O)=C(S(NC2=CC=C(C(O)=O)C=C2)(=O)=O)C=C1	29 ± 3	19 ± 2
**DSF085**	ClC1=CC([N+]([O-])=O)=C(S(NC2=CC=C(C(O)=O)C(O)=C2)(=O)=O)C=C1	36 ± 5	0 ± 4
**DSF086**	ClC1=CC([N+]([O-])=O)=C(S(NC2=CC=CC(Cl)=C2)(=O)=O)C=C1	19 ± 4	0 ± 3
**DSF087**	ClC1=CC([N+]([O-])=O)=C(S(NC2=CC=C(C(OCC)=O)C=C2)(=O)=O)C=C1	36 ± 4	2 ± 3
**DSF088**	ClC1=CC([N+]([O-])=O)=C(S(NC2=CC(OC)=CC=C2OC)(=O)=O)C=C1	22 ± 3	0 ± 3
**DSF089**	ClC1=CC([N+]([O-])=O)=C(S(N2CCOCC2)(=O)=O)C=C1	22 ± 4	20 ± 5
**DSF090**	ClC1=CC([N+]([O-])=O)=C(S(N2CCCCC2)(=O)=O)C=C1	19 ± 1	12 ± 2

Supplementary Bibliography

[22] Zaryanova, E. V.; Lozinskaya, N. A.; Beznos, O. V.; Volkova, M. S.; Chesnokova, N. B.; Zefirov, N. S. Oxindole-based intraocular pressure reducing agents. *Bioorg Med Chem Lett* **2017**, *27* (16), 3787-3793. DOI: 10.1016/j.bmcl.2017.06.065.

[47] Palluotto, F.; Campagna, F.; Carotti, A.; Ferappi, M.; Rosato, A.; Vitali, C. Synthesis and antibacterial activity of pyridazino[4,3-b]indole-4-carboxylic acids carrying different substituents at N-2. *Farmaco* **2002**, *57*, 63-69. DOI: 10.1016/s0014-827x(01)01173-9.

[48] Jin, K.; Zhang, X.; Ma, C.; Xu, Y.; Yuan, Y.; Xu, W. Novel indoline-2,3-dione derivatives as inhibitors of aminopeptidase N (APN). *Bioorg Med Chem* **2013**, *21* (9), 2663-2670. DOI: 10.1016/j.bmc.2012.06.024.

[50] Daina, A.; Michielin, O.; Zoete, V. SwissADME: a free web tool to evaluate pharmacokinetics, drug-likeness and medicinal chemistry friendliness of small molecules. *Sci Rep* **2017**, *7*, 42717. DOI: 10.1038/srep42717.

[62] Acquasaliente, L.; Peterle, D.; Tescari, S.; Pozzi, N.; Pengo, V.; De Filippis, V. Molecular mapping of α-thrombin (αT)/β2-glycoprotein I (β2GpI) interaction reveals how β2GpI affects αT functions. *Biochem J* **2016**, *473* (24), 4629-4650. DOI: 10.1042/BCJ20160603.
